# Base editing repairs an *SGCA* mutation in human primary muscle stem cells

**DOI:** 10.1172/jci.insight.145994

**Published:** 2021-05-24

**Authors:** Helena Escobar, Anne Krause, Sandra Keiper, Janine Kieshauer, Stefanie Müthel, Manuel García de Paredes, Eric Metzler, Ralf Kühn, Florian Heyd, Simone Spuler

**Affiliations:** 1 Muscle Research Unit, Experimental and Clinical Research Center, a cooperation between the Max-Delbrück-Center for Molecular Medicine in the Helmholtz Association and the Charité, Universitätsmedizin Berlin, Germany.; 2 Charité Universitätsmedizin Berlin, Germany.; 3 Max-Delbrück-Center for Molecular Medicine in the Helmholtz Association, Berlin, Germany.; 4 Freie Universität Berlin, Institute of Chemistry and Biochemistry, Laboratory of RNA Biochemistry, Berlin, Germany.

**Keywords:** Stem cells, Therapeutics, Human stem cells, Monogenic diseases, Skeletal muscle

## Abstract

Skeletal muscle can regenerate from muscle stem cells and their myogenic precursor cell progeny, myoblasts. However, precise gene editing in human muscle stem cells for autologous cell replacement therapies of untreatable genetic muscle diseases has not yet been reported. Loss-of-function mutations in *SGCA*, encoding α-sarcoglycan, cause limb-girdle muscular dystrophy 2D/R3, an early-onset, severe, and rapidly progressive form of muscular dystrophy affecting both male and female patients. Patients suffer from muscle degeneration and atrophy affecting the limbs, respiratory muscles, and heart. We isolated human muscle stem cells from 2 donors, with the common *SGCA* c.157G>A mutation affecting the last coding nucleotide of exon 2. We found that c.157G>A is an exonic splicing mutation that induces skipping of 2 coregulated exons. Using adenine base editing, we corrected the mutation in the cells from both donors with > 90% efficiency, thereby rescuing the splicing defect and α-sarcoglycan expression. Base-edited patient cells regenerated muscle and contributed to the Pax7^+^ satellite cell compartment in vivo in mouse xenografts. Here, we provide the first evidence to our knowledge that autologous gene–repaired human muscle stem cells can be harnessed for cell replacement therapies of muscular dystrophies.

## Graphical abstract

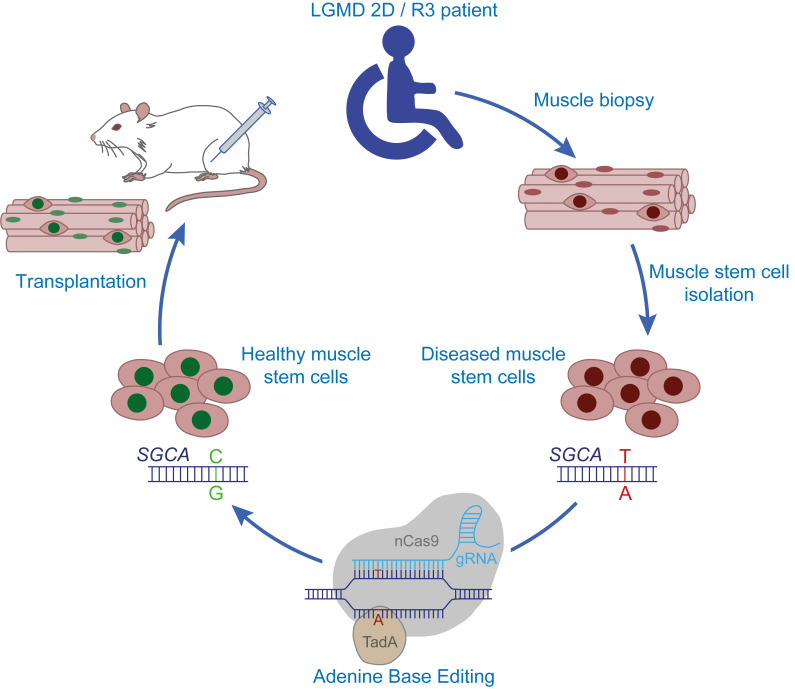

## Introduction

Limb-girdle muscular dystrophies (LGMD) include almost 30 different monogenic diseases characterized by progressive weakness and atrophy presenting in the shoulder and pelvic girdle muscles. The total prevalence is 2.19 per 100,000 (95% CI, 1.78–2.70) (1). There is no therapy. One of the most severe and frequent types is caused by α-sarcoglycan deficiency due to mutations in *SGCA* (classified as LGMD2D or LGMDR3). Affected patients become increasingly week in the late first decade of life and often become wheelchair-bound during puberty. Cardiac and respiratory involvement are possible. *SGCA* has 9 coding exons and is expressed in striated muscle (2, 3). Disease-causing mutations are spread along the entire length of the gene without defined mutational hot spots (4, 5). However, some mutations like c.157G>A have been reported more frequently (6, 7). α-Sarcoglycan is a 50 kDa transmembrane protein, part of the sarcoglycan complex and the dystrophin-associated protein complex (DAPC) (2, 3). The DAPC protects muscle fibers from mechanical stress, and its dysfunction leads to various forms of muscular dystrophy (MD) (8, 9).

Muscle fibers are syncytial structures with postmitotic nuclei formed by the fusion of myogenic progenitor cells, called myoblasts, during prenatal and postnatal development. Skeletal muscle can regenerate from muscle stem cells (MuSC), also called satellite cells, a pool of tissue-specific stem cells located between the muscle fiber membrane (sarcolemma) and the basal lamina that surrounds every fiber (10). In healthy muscle, satellite cells are quiescent or slow cycling. When activated in response to severe damage, they extensively proliferate and give rise to large numbers of myoblasts that fuse to damaged myofibers or to one another to generate new myofibers (11). Skeletal muscle regeneration cannot occur without satellite cells (12, 13). Patients with MD suffer constant tissue degeneration, which prompts satellite cells to be constantly activated, leading to satellite cell exhaustion, regenerative deficit, and replacement of muscle by fat and connective tissue (14, 15).

Cell replacement therapies with well-defined and highly myogenic cell populations could represent a safe and long-term treatment avenue for MD patients (16); however, MuSC are scarce and difficult to manipulate ex vivo. In addition, skeletal muscle is the most abundant tissue in the body; therefore, developing cell replacement therapies for MD patients poses substantial challenges. We have previously shown that MuSC can be isolated and substantially expanded from human muscle biopsy specimens and that they maintain in vivo regenerative capacity in xenograft models (17, 18). Using them in an autologous setting for MD patients would require correcting the genetic defect before reimplantation.

Precise and efficient gene repair in primary somatic stem and progenitor cells ex vivo is increasingly plausible due to the rapid development of CRISPR/Cas9-based tools for base editing that are independent from the cellular DNA repair pathway choice. Adenine base editing (ABE) enables the precise targeted conversion of adenine into guanine nucleotides without inducing DNA double-strand breaks (19). An ABE consists of a catalytically impaired Cas9 in fusion with an adenine deaminase enzyme (TadA) that converts adenine into inosine on the single-stranded DNA bubble created by Cas9 binding to a target site. Inosine is subsequently replaced by guanine. To be accessible to the deaminase, the target adenine must be located at a defined distance from the protospacer adjacent motif (PAM), the so-called ABE activity window (19). Because of its predictable outcome, high precision and reduced off-target effects (20, 21), ABE is potentially the safest gene editing tool to date. However, despite technical advances in tool development, therapeutic gene or base editing in clinically relevant human MuSC has not yet been reported.

Here, we found a potentially new pathomechanism for a loss-of-function *SGCA* c.157G>A mutation and corrected it with > 90% efficiency in primary MuSC from a LGMD2D patient and a related carrier using ABE, without detectable editing at predicted off-target loci. ABE-corrected patient MuSC functionally engrafted and reconstituted the satellite cell compartment following intramuscular transplantation in a xenograft model. We hereby provide the first evidence to our knowledge that primary human MuSC can be efficiently and safely gene edited and harnessed for autologous cell replacement therapies of MD.

## Results

### Isolation of primary MuSC from a patient and a carrier with a compound heterozygous SGCA c.157G>A mutation.

**Figure 1:**
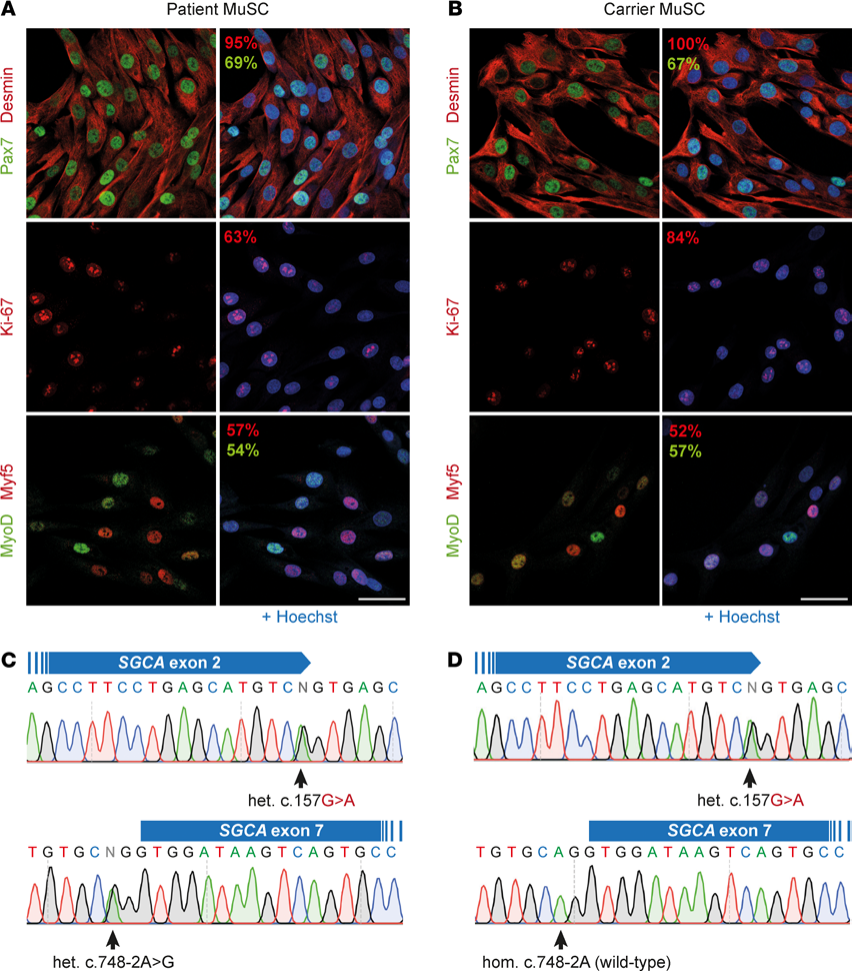
Characterization of primary MuSC from 2 donors with a heterozygous *SGCA* c.157G>A mutation. (**A** and **B**) Immunostaining for the satellite cell marker Pax7; the myogenic markers Desmin, MyoD, and Myf5; and the proliferation marker Ki-67 in patient (**A**) and carrier (**B**) primary MuSC cultures. The percentage of cells expressing each marker is displayed on the images in the corresponding colors. Nuclei were counterstained with Hoechst. At least 100 nuclei were counted per sample, and staining was done before cryopreservation and after thawing. (**C**) *SGCA* sequence analysis from the patient shows the compound heterozygous mutation in exon 2 (c.157G>A) and the splice acceptor of exon 7 (c.748-2A>G). (**D**) *SGCA* sequence analysis from the carrier shows the compound heterozygous mutations in exon 2 (c.157G>A) and a homozygous WT exon 7 sequence. Scale bars: 50 μm.

We isolated and characterized primary MuSC from muscle biopsy specimens obtained from a 10-year-old male LGMD2D patient carrying a compound heterozygous *SGCA* c.157G>A mutation and from a related carrier (Figure 1, A–D). Primary MuSC cultures from patient and carrier were 95%–100% Desmin^+^ and expressed the myogenic markers Pax7, MyoD, and Myf5 and the proliferation marker Ki-67 (Figure 1, A and B). The c.157G>A mutation affects the last coding nucleotide of exon 2 (Figure 1, C and D). Our patient has a second heterozygous loss-of-function *SGCA* mutation (c.748-2A>G) in the splice acceptor of exon 7 (Figure 1C). We also obtained primary MuSC from a related carrier of the c.748-2A>G mutation (Supplemental Figure 1; supplemental material available online with this article; https://doi.org/10.1172/jci.insight.145994DS1).

### SGCA c.157G>A is an exonic splicing mutation.

**Figure 2:**
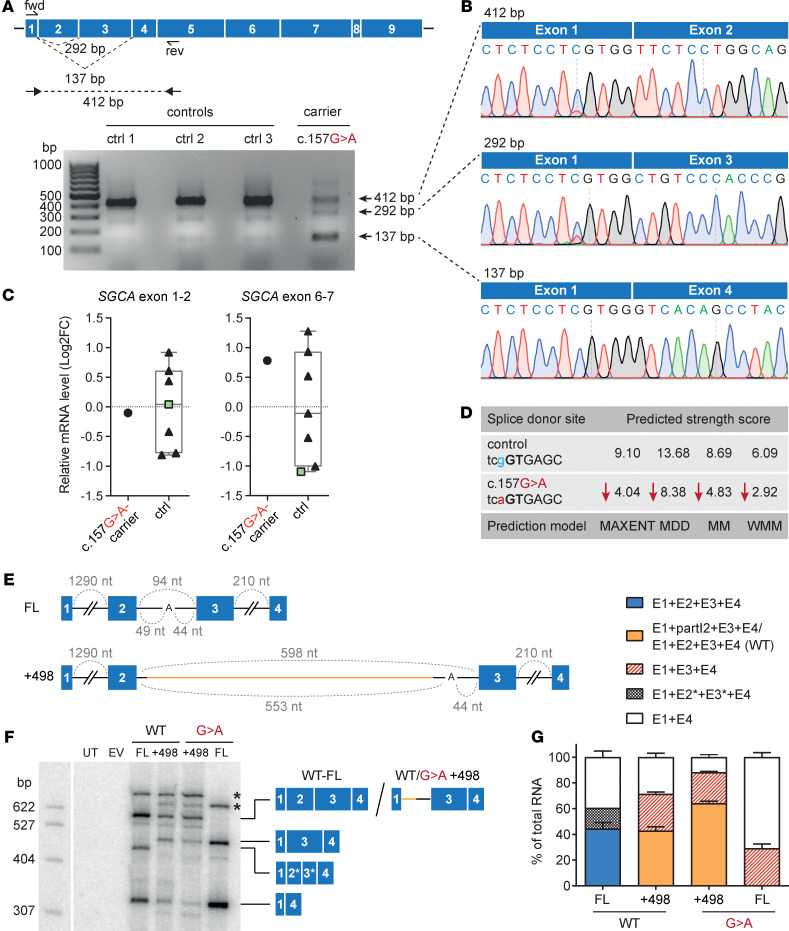
*SGCA* c.157G>A is an exonic splicing mutation. (**A**) RT-PCR analysis of *SGCA* mRNA in muscle tissue from controls and the c.157G>A carrier. Primer binding sites and expected band sizes are displayed in the panel above. Control 3 is a heterozygous *SGCA* c.748-2A>G carrier with a WT *SGCA* exon 2 sequence. The RT-PCR was performed 3 times. (**B**) Sequencing of the bands from **A** shows skipping of exon 2 or exon 2+3 in the c.157G>A carrier. (**C**) qPCR analysis of *SGCA* mRNA in muscle tissue from controls and the c.157G>A carrier using primers against the exon 1–2 or exon 6–7 boundaries. Control 3 is represented by a green square. The qPCR was performed in technical triplicates. Values were normalized to *GAPDH* and relativized to the mean of controls. (**D**) Strength scores of the *SGCA* exon 2 splice donor for the WT and the mutant sequence predicted by MaxEntScan:score5ss. (**E**) Minigene construct schemes. FL, full-length *SGCA* exon 1–4 (blue boxes) with the intermediate introns (black lines); +498, a 498 bp-long low complexity intronic sequence from the human *HBB* gene (yellow) was inserted to extend intron 2. The size of each intron is indicated above in gray. For intron 2, the size before and after the branch point is indicated below in gray. UT, untransfected; EV, empty vector; WT, WT *SGCA*; G>A, c.157G>A mutation. (**F**) Minigene splicing patterns in HEK293T cells analyzed by low-cycle RT-PCR with a ^32^P-labeled forward primer; products were separated by denaturing PAGE. The splice isoforms identified are shown on the right (identity confirmed by Sanger sequencing). *, intron-retention isoforms; 2*, truncated exon 2 (40 nt); 3*, truncated exon 3 (–70 nt). (**G**) Splice isoform quantification from **F** using Phosphorimager analysis. Quantified values are presented as mean ± SD (*n* = 3). E, exon; I, intron.

*SGCA* c.157G>A has been reported as a missense variant (p.Ala53Thr) (6, 7), but its consequences on mRNA level were not investigated. We analyzed the *SGCA* mRNA and found 2 smaller bands corresponding to the skipping of exon 2 alone or exons 2 and 3 in the heterozygous c.157G>A carrier (Figure 2, A and B). No c.157A was detectable in the band corresponding to the full-length mRNA, indicating that residual inclusion of the mutant exon 2 is negligible (Supplemental Figure 2A; see complete unedited blots in the supplemental material). Splicing of exons 4–9 was unaffected (Supplemental Figure 2B). Overall, there was no significant difference in the relative *SGCA* mRNA levels between carrier and controls (Figure 2C). We then analyzed the predicted strength of the 5′ splice site of *SGCA* exon 2 using MaxEntScan:score5ss (22). We found that the mutation strongly decreases the strength of the 5′ splice site (Figure 2D). These results indicate that *SGCA* c.157G>A is an exonic splicing mutation.

To better understand the mechanism underlying the codependent splicing of exons 2 and 3, we designed *SGCA* WT and c.157G>A minigene constructs covering exons 1–4 and the interjacent introns 1–3 (Figure 2E). Splice sites can be recognized by the spliceosome in a cross-intron complex (intron definition) or in a cross-exon complex (exon definition). Exon definition happens if exons are flanked by long introns, as is the case for most human exons, and the initial exon-defined complex must be transferred into an intron-defined complex for productive splicing. *SGCA* exons 2 and 3 are separated by a short intron of 94 nt. Splice-site recognition for short introns (<200–250 nt) occurs via the intron definition pathway and is more efficient, resulting in enhanced inclusion of exons with weak splice sites (23). Thus, we reasoned that the short length of intron 2 could contribute to the efficient splicing of both exons 2 and 3. Extending the length of intron 2 should induce a switch from intron to exon definition, thereby reducing the efficiency of exon 2 and 3 splicing and making exon 3 inclusion independent of the c.157G>A mutation. To test this hypothesis, we additionally created minigene constructs where intron 2 was extended by 498 nt using a low complexity intronic sequence from the human β-globin gene (Figure 2E).

We analyzed splicing of these minigenes in HEK293T cells and observed similar patterns for the WT and c.157G>A *SGCA* constructs as observed in human muscle tissue from controls or the heterozygous carrier, respectively (Figure 2, F and G; Supplemental Figure 2C; and Supplemental Figure 3). The full-length mRNA isoform including exons 1–4 was completely abolished by the c.157G>A mutation, which resulted in skipping of exon 2 alone or, more frequently, in skipping of exons 2 and 3. Consistent with our model, extending intron 2 induced exon 2 skipping independently of the c.157G>A mutation, which was frequently accompanied by exon 3 skipping. A weak cryptic splice site within the extended intron was occasionally selected, resulting in the inclusion of exon 3 and a segment of intron 2 (Figure 2, F and G, and Supplemental Figure 3). These splicing patterns suggest that intron definition of the short intron 2 is required for the correct recognition of both exons 2 and 3, and they provide a mechanistic explanation for the effect of the c.157G>A mutation on exon 3 splicing.

### ABE corrects the SGCA c.157G>A mutation in patient-derived induced pluripotent stem cells.

**Figure 3:**
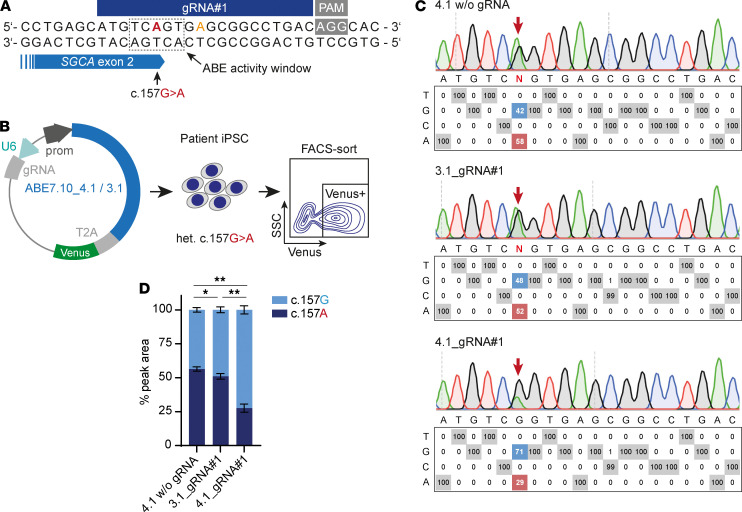
ABE corrects the *SGCA* c.157G>A mutation in patient-derived iPSC. (**A**) c.157G>A is located in the center of the ABE activity window for gRNA#1. (**B**) Experimental design. A plasmid encoding ABE7.10_4.1 or ABE7.10_3.1 was transfected into patient iPSC. Venus^+^ cells were selected via FACS, and the bulk sorted population was analyzed. (**C**) EditR analysis of nucleotide rates at each protospacer position in patient iPSC transfected with ABE7.10_4.1 and _3.1 in combination with gRNA#1. iPSC transfected with ABE7.10_4.1 without gRNA are shown as control. (**D**) c.157G/A nucleotide rates from EditR analysis. Quantified values are presented as mean ± SD (*n* = 5). Statistical analysis of the difference in c.157G nucleotide rates (light blue) between the columns was performed using the Mann-Whitney *U* test. Two-tailed *P* values were calculated. *P < 0.02; **P < 0.01.

To work out strategies to genetically correct the c.157G>A mutation, we generated induced pluripotent stem cells (iPSC) from the patient (Supplemental Figure 4). We identified c.157G>A as an ideal ABE target, since it is located 15 bp upstream of an –NGG PAM (equivalent to protospacer position 6, thus in the center of the ABE activity window). No other adenines are located within the ABE activity window, so undesired bystander edits are unlikely (Figure 3A). We first assessed if ABE can be used to repair the c.157G>A mutation in patient iPSC. We transfected these cells with a plasmid encoding ABE7.10_4.1, a vector based on ABE7.10 (19) containing a codon-optimized Cas9 (D10A) nickase N-terminally fused to the TadA heterodimer, followed by a T2A-Venus cassette under control of the CAG promoter, and an sgRNA expression cassette. We enriched for Venus^+^ cells via FACS (Figure 3B and Supplemental Figure 5B) and assessed ABE efficiency using EditR (24). We found that ABE7.10_4.1 induced efficient c.157A>G conversion when combined with a suitable gRNA (gRNA#1), without any detectable bystander A>G edits (Figure 3, C and D, and Supplemental Figure 6). We additionally examined the ability of the enhanced specificity Cas9 variant eSpCas9(1.1) (25), fused to the TadA heterodimer in an identical configuration (ABE7.10_3.1), to induce A>G conversions at this locus but detected only minimal editing. We thus selected ABE7.10_4.1 for further experiments.

### ABE results in > 90% correction of SGCA c.157G>A in primary human MuSC without detectable off-target editing.

**Figure 4:**
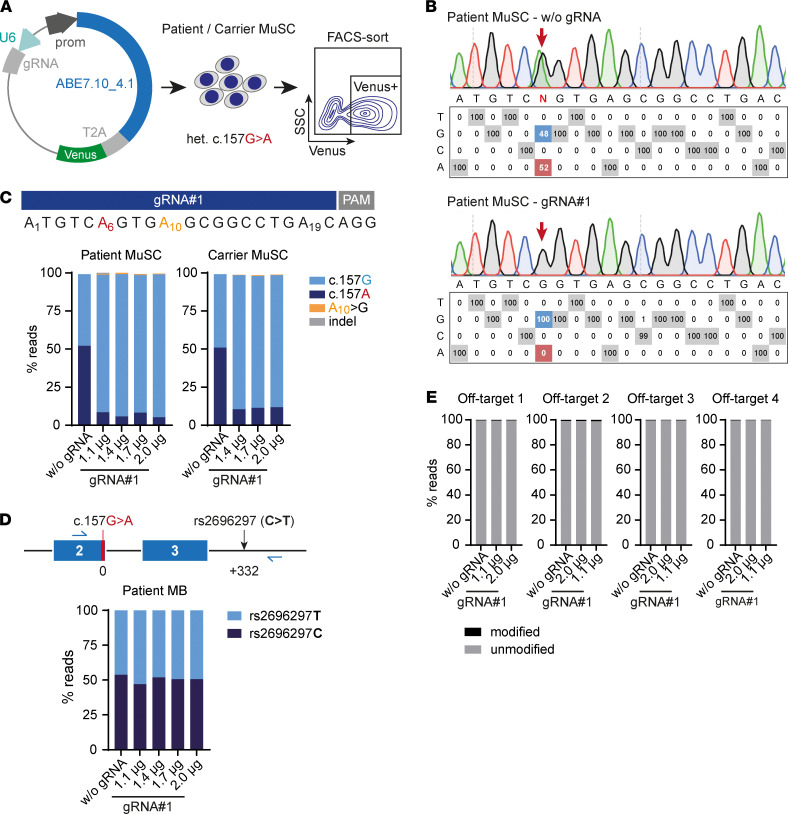
ABE repairs the *SGCA* c.157G>A mutation in patient and carrier primary MuSC without detectable off-target editing. (**A**) Experimental design. Primary MuSC from the patient and the carrier were transfected with ABE7.10_4.1/gRNA#1 or without gRNA. FACS was performed to enrich for Venus^+^ cells, which were subsequently analyzed. (**B**) EditR analysis of nucleotide rates at each protospacer in patient MuSC transfected with ABE7.10_4.1/gRNA#1. (**C**) Percentage of reads containing c.157G/A, bystander editing of A_10_, and indels in patient and carrier MuSC transfected with a range of ABE7.10_4.1/gRNA#1 vector concentrations. (**D**) A SNP located 332 bp downstream of the mutation and heterozygous in the patient was included in the amplicon to rule out allele detection bias. The plot shows the percentage of reads aligned to each allele. (**E**) Amplicon sequencing analysis of the 4 predicted exonic off-target sites. Amplicon sequencing data were analyzed using CRISPResso2.

We next asked if ABE could efficiently repair the c.157G>A mutation and rescue the phenotype in human primary MuSC. We transfected MuSC from the patient and the heterozygous carrier with various concentrations of the ABE7.10_4.1/gRNA#1 vector, as above, and enriched for Venus^+^ cells (Figure 4A and Supplemental Figure 5A). Following sorting, we expanded the Venus^+^ cells in culture and analyzed on- and off-target editing. All vector concentrations resulted in > 99% c.157G nucleotide rates in patient and carrier MuSC as analyzed by EditR (Figure 4B). We then performed amplicon sequencing with subsequent analysis by CRISPResso2 (26) and confirmed high c.157G nucleotide rates of > 90% for patient MuSC and > 85% for carrier MuSC (Figure 4C, Supplemental Figure 7, and Supplemental Figure 8). Bystander A>G editing at protospacer position 10 was detected in a very low (0.2%–2%) percentage of reads. In 2 samples, we detected 1.1% and 0.3% of reads containing indels. Omission of gRNA did not result in either A>G editing or indels (Figure 4C, Supplemental Figure 7, and Supplemental Figure 8). To rule out allele-detection bias, we designed a PCR amplicon that includes a heterozygous single nucleotide polymorphism (SNP) located 332 bp downstream of c.157G>A in the patient *SGCA* gene (SNP ID: rs2696297). We confirmed an equal representation of both alleles in our amplicon sequencing data, thus ruling out detection bias (Figure 4D). This analysis was not possible for the carrier MuSC, where this SNP is homozygous. We next aimed to characterize the off-target profile of our editing approach. We investigated the 4 predicted exonic off-target sites that contain adenines within the ABE activity window via amplicon sequencing and CRISPResso2 analysis. We could not detect any Cas9-dependent off-target editing events at these loci with either the lowest or highest vector concentration (Figure 4E, Supplemental Figure 9, and Supplemental Figure 10). Our detection threshold for reads containing off-target events ranged from 0.01% for the locus with the lowest number of aligned reads per sample (*ZNF571*; Supplemental Figure 10A) to 0.0004% for the locus with the highest number of aligned reads per sample (*KIF1A*; Supplemental Figure 10B). We thus have concluded that the *SGCA* c.157G>A mutation can be repaired in human primary MuSC with very high efficiency and specificity via ABE. Repaired *SGCA* c.157G>A is hereafter referred to as *SGCA* c.157Grep.

### SGCA c.157Grep primary MuSC show normal α-sarcoglycan mRNA and protein expression.

**Figure 5:**
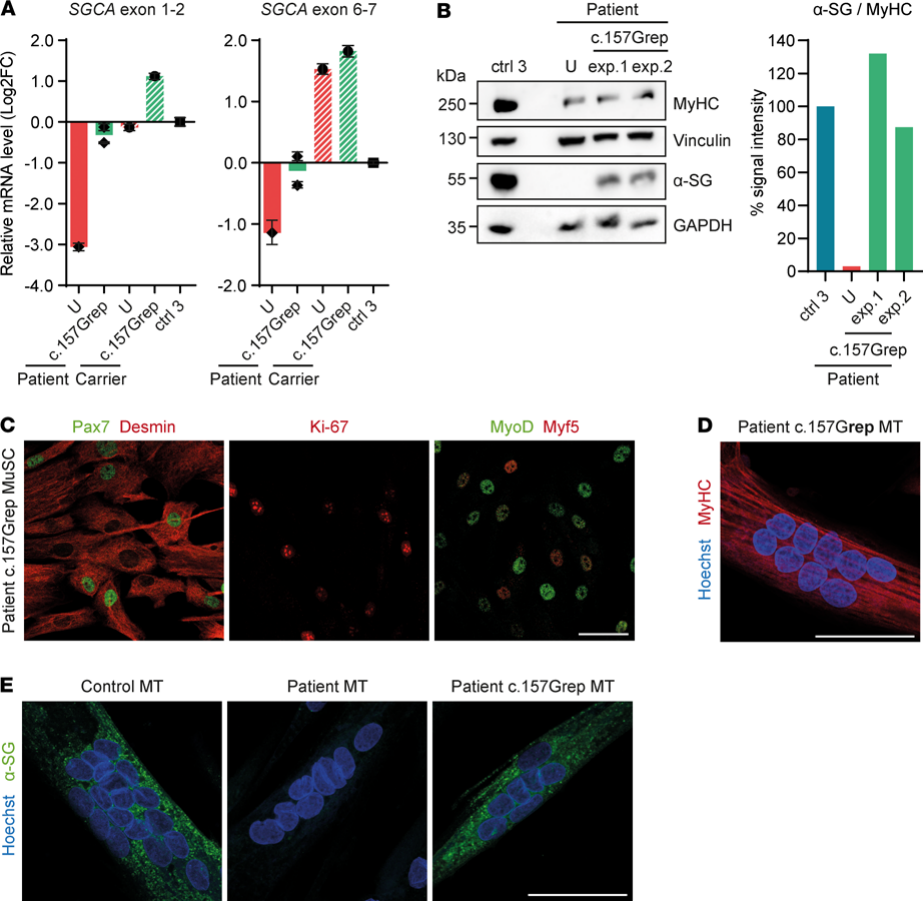
*SGCA* c.157Grep patient MuSC express α-sarcoglycan, and they are viable and myogenic. (**A**) qPCR analysis of *SGCA* mRNA in *SGCA* c.157Grep compared with unedited (U) patient and carrier myotubes. Values are normalized to *GAPDH* and relativized to control 3. *SGCA* mRNA values were additionally normalized to *MYH2* to correct for differences in the differentiation stage of the cells, which may affect promoter activity. Each data point represents the mean ± SD for the *n* = 3 technical replicates of each biological sample. (**B**) Western blot analysis of α-sarcoglycan protein in *SGCA* c.157Grep patient myotubes compared with unedited (U) patient and control 3 myotubes. Myosin heavy chain (MyHC) was used as a differentiation marker. Vinculin and GAPDH were used as loading controls. The intensity of α-sarcoglycan Western blot bands was quantified using ImageJ and normalized to Vinculin and MyHC. (**C**) *SGCA* c.157Grep patient MuSC were immunostained for Pax7, Desmin, MyoD, Myf5, and Ki-67. The analysis was performed for all *SGCA* c.157Grep patient cell populations, and a quantification is shown in Supplemental Table 1. (**D**) *SGCA* c.157Grep MuSC readily fused into multinucleated myotubes expressing MyHC. The analysis was performed for all *SGCA* c.157Grep patient cell populations. (**E**) α-Sarcoglycan immunostaining in control myotubes, as well as unedited and *SGCA* c.157Grep patient myotubes. The analysis was performed for all *SGCA* c.157Grep patient cell populations. MT, myotubes. Scale bars: 50 μm.

To assess the functional outcome of ABE, we analyzed α-sarcoglycan mRNA and protein expression in *SGCA* c.157Grep myotubes. We found that the splicing defect was rescued as shown by the increase in α-sarcoglycan transcripts containing exon 2 in *SGCA* c.157Grep compared with unedited patient and carrier myotubes, reaching levels similar to control 3 (heterozygous c.748-2A>G carrier) in the case of patient myotubes (Figure 5A). Furthermore, total *SGCA* mRNA levels increased in patient myotubes following ABE (Figure 5A), probably because coskipping of exons 2 and 3 (but not exon 2 alone) induces a frameshift, leading to a premature stop codon, which could result in nonsense-mediated mRNA decay (NMD). Western blot and immunostaining analysis revealed that α-sarcoglycan protein was restored in *SGCA* c.157Grep patient cells (Figure 5, B and E).

### SGCA c.157Grep primary patient MuSC are viable, proliferative, and myogenic.

Primary cells are especially susceptible to stress induced by extensive manipulation. Primary MuSC derived from MD patients with mutations in genes responsible for membrane integrity are particularly vulnerable. We observed a decrease in cell proliferation in the first days following transfection and sorting as compared with untransfected patient MuSC. However, Venus^+^ cells (≥48% of the source cell population; Supplemental Figure 5A) proliferated extensively after sorting and were further expanded for at least 2–3 passages before cryopreservation. Samples for on- and off-target editing analysis were collected at this time point. We assessed Desmin, Pax7, MyoD, Myf5, and Ki-67 expression in all *SGCA* c.157Grep patient MuSC populations at the time of cryopreservation. We found that, in unedited patient MuSC, the percentage of Desmin^–^ cells increased with the passages up to approximately 11%. All but 1 *SGCA* c.157Grep patient MuSC populations had > 98% Desmin^+^ cells. Myogenic marker expression was comparable in *SGCA* c.157Grep pure MuSC populations and passage-matched unedited patient MuSC (Figure 5C and Supplemental Table 1). *SGCA* c.157Grep MuSC were recovered and further expanded for 1 passage before transplantation, at which time the expression of myogenic and proliferation markers was reassessed. The percentage of cells positive for those markers was maintained (Supplemental Table 1). *SGCA* c.157Grep primary MuSC could readily fuse into multinucleated myotubes in vitro (Figure 5D). Moreover, the pattern of α-sarcoglycan localization was indistinguishable from control myotubes (Figure 5E).

### SGCA c.157Grep primary MuSC regenerate muscle and repopulate the satellite cell niche in vivo.

**Figure 6:**
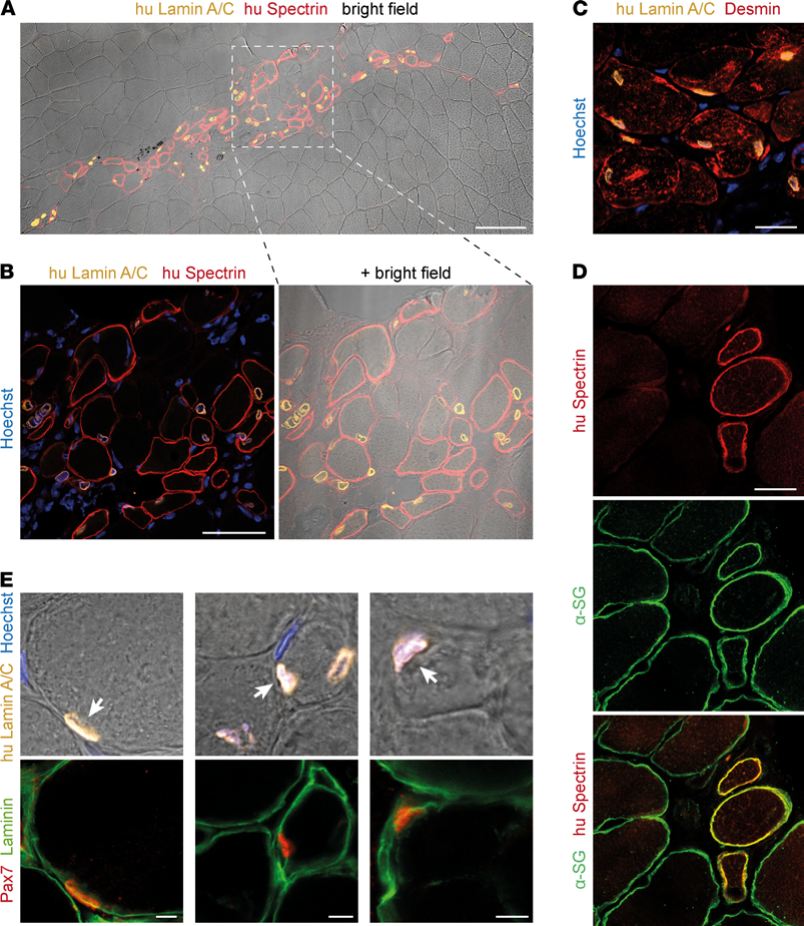
*SGCA* c.157Grep patient MuSC regenerate muscle in vivo. (**A**) *SGCA* c.157Grep patient MuSC were injected into preirradiated anterior tibial muscles of immunocompromised NSG mice. Grafted muscles were collected for analysis after 19 days. (**A** and **B**) Grafted muscles were immunostained with antibodies that specifically recognize human Lamin A/C and human Spectrin, labeling donor nuclei and donor-derived myofibers, respectively. In total, 31–87 human nuclei and 33–72 human myofibers were found per section (*n* = 2). (**C**) Grafted muscles were immunostained with antibodies against human Lamin A/C and Desmin. (**D**) Grafted muscles were immunostained with antibodies against human Spectrin and α-sarcoglycan. (**E**) Grafted muscles were immunostained with antibodies against human Lamin A/C, Pax7, and Laminin to identify satellite cells of human origin located in the stem cell niche under the basal lamina (*n* = 2). Human Pax7^+^ satellite cells are indicated by arrows. Nuclei were counterstained with Hoechst. Scale bars: 100 μm (**A**), 50 μm (**B**), 20 μm (**C** and **D**), 5 μm (**E**).

We next asked if *SGCA* c.157Grep primary MuSC would contribute to muscle regeneration in vivo. We, thus, transplanted them into irradiated anterior tibial muscles of immunocompromised NSG mice. We found that *SGCA* c.157Grep patient MuSC gave rise to human muscle fibers (Figure 6, A–C) that expressed α-sarcoglycan (Figure 6D and Supplemental Figure 11). Furthermore, the satellite cell niche between the sarcolemma and the basal lamina was populated with numerous Pax7^+^ cells of human origin (Figure 6E). Taken together, *SGCA* c.157Grep patient MuSC are capable of both myofiber regeneration and reconstitution of the satellite cell compartment in vivo.

## Discussion

Therapeutic gene editing in muscular dystrophies is developing into a realistic scenario. The exact treatment regimen will depend on the affected muscles, the diseased gene, and the type of mutation. Gene supplementation therapy using adeno-associated viral (AAV) vectors may be suitable for genes up to a certain size. However, the exogenously provided cDNA is not physiologically regulated in terms of splicing and spaciotemporal expression and poses a risk of insertional mutagenesis. AAV-mediated CRISPR/Cas9 delivery directly into the muscle has enabled highly efficient in vivo gene editing in Duchenne muscular dystrophy (DMD) mice and large animals models (27–30), and it could potentially reach and permanently repair a very large fraction of myonuclei. In vivo ABE in muscle has been achieved in a DMD mouse model (31). However, if not done before substantial degeneration, fatty-fibrous replacement, or satellite cell exhaustion have occurred, the disease course may not be reversible. In addition, liver toxicity associated with systemic AAV administration has resulted in fatal complications in some patients who received the highest viral dose in a gene supplementation clinical trial for myotubular myopathy, calling for very cautious planning in future trials of systemic AAV administration (32). All of this has put stem cell therapies back on stage in the treatment of muscular dystrophies (16).

We, for the first time to our knowledge, demonstrate here highly efficient and precise correction of a MD-causing mutation in primary patient MuSC using ABE. The edited cells maintain their ability to produce new muscle fibers and repopulate the Pax7^+^ stem cell pool in vivo as shown previously for healthy human MuSC (17, 18). These cells are transplantable in an autologous setting with a small and calculable risk, and the repaired gene sequence maintains the endogenous regulation machinery in place. Although the availability of primary human MuSC in early passages and with high regenerative capacity is limited, this should not preclude further developments for several reasons. First, minimizing stress during gene repair and cell expansion to obtain large numbers of repaired cells is a challenging but solvable problem. Human MuSC expansion on improved scaffolds has resulted in high proliferation rates (33), and culturing human MuSC in niche-like environments has been shown to enhance their engraftment potential (17, 34). In the case of MD patients, the time at which the muscle biopsy for MuSC isolation is obtained may be crucial and should be carefully considered. Second, although protocols to differentiate iPSC into cells with myogenic potential in xenograft models have been developed (35, 36, 37), the derived cells so far lack purity and maturity. Nevertheless, iPSC-derived myogenic cells have successfully been used to model LGMD 2D/R3 in vitro (38) and could become a relevant source for cell replacement therapies in the future, provided that their safety and efficacy profile matches the requirements for clinical use. Third, the reconstitution of single muscles that could be achieved with limited cell numbers may well result in improved patient autonomy and life quality. Examples would be transplantation into finger flexors, resulting in improved grip strength or restored strength of respiratory muscles, such as the diaphragm. Similarly, swallowing function was improved in oculopharyngeal muscular dystrophy (OPMD) patients following transplantation of autologous nongenetically corrected myoblasts into the pharyngeal muscles. However, a long-term therapeutic effect would likely require transplanting gene-corrected autologous MuSC (39).

Compared with classical CRISPR/Cas9-based approaches that rely on the cellular DNA repair machinery to install edits, base editing does not require DNA double-strand breaks and has a predictable, well-defined, and precise repair outcome (19). It is also cell-cycle independent and, thus, suitable for editing in postmitotic syncytial myonuclei and MuSC. Sequence restrictions like PAM requirements and the presence of other editable nucleotides in the activity window currently limit the scope of base editors. However, the fast-expanding landscape of new Cas enzymes with widened PAM specificities and the latest generation deaminase domains (40–42) are rapidly widening the spectrum of mutations amenable to base editing. A question that remains open is how to reconcile faster kinetics and wider editing scopes with safe off-target profiles, especially for therapeutic applications. Here, we used ABE7.10, which has been shown to produce minimal Cas9-dependent or -independent DNA and RNA off-target editing in vitro and in vivo (20, 21, 40, 41). Bystander A>G editing at protospacer position 10 was detectable in *SGCA* c.157Grep MuSC, albeit at very low levels. Its functional consequences are difficult to predict because this adenine is in intron 2. Notably, another MD-causing *SGCA* G>A mutation is located just 1 nucleotide downstream (c.157+1G>A) (5, 43), within the same ABE activity window as shown here, and it could presumably be corrected with an almost identical approach.

We could not detect ABE-induced Cas9–dependent editing at any of the predicted exonic off-target loci. For the gRNA used in this study, all predicted off-target sites have at least 3–4 mismatches to the target sequence. Furthermore, all off-target sites except 1 contain several mismatches within the seed sequence (nucleotides 1–12 proximal to the PAM), which makes them unlike targets for Cas9 binding. While we cannot rule out Cas9-dependent or -independent off-target editing in other loci, we found no evidence of malignant proliferation of *SGCA* c.157Grep MuSC in vitro or following transplantation into NSG mice. For therapeutic purposes, we propose a targeted analysis of predicted off-target sites, proto-oncogenes, and tumor-suppressor genes. Whole genome sequencing could reveal off-target events in unpredicted loci, but low-frequency sequence alterations may be difficult to detect.

Although most proof-of-concept gene editing studies focus on homozygous mutations or X-linked diseases like DMD, we argue that this is hardly a realistic scenario for diseases like LGMD, where the vast majority of patients have heterozygous mutations (5). In patients with autosomal recessive LGMD, repairing 1 allele would convert the cells into a carrier-like genotype, thus effectively restoring the phenotype. Most patients with > 30% α-sarcoglycan levels remain ambulant until age 60 as opposed to < 20 years for patients with completely absent protein (5), evidencing that increasing the levels of α-sarcoglycan in the muscle to even less than 50% could dramatically slow down disease progression.

A particular challenge of repairing heterozygous mutations, whereby the repaired sequence is identical to the WT sequence, is potential allele detection bias resulting in a wrong interpretation of gene editing outcomes (44, 45). Although ABE requires that only 1 DNA strand is nicked, large indels that interfere with primer binding sites cannot be excluded and may be especially relevant in the context of therapeutic cell products. Here, we have included a heterozygous SNP in the same amplicon as the edit to unequivocally rule out an unbalanced allele representation in our data set.

For all monogenic diseases like MD, understanding the pathomechanism of disease-associated variants is crucial for proper patient stratification and to administer or develop the right therapies. Numerous MD-causing mutations classified as missense result in complete loss of protein. *SGCA* c.157G>A was initially described as a loss-of-function missense variant leading to a complete absence of α-sarcoglycan (6, 7). Here, we describe a splicing defect as the main consequence of this mutation. Although it does not disrupt the reading frame, exon 2 skipping removes > 10% of the *SGCA* coding sequence. An intriguing finding is the coskipping of exons 2 and 3, which is enhanced by the mutation and induces a frameshift. We suggest that recognition of the short *SGCA* intron 2 by the spliceosome through the intron definition pathway serves as a mechanism to promote the coregulated inclusion of exons 2 and 3. This conclusion is based on our finding that impairment of intron recognition induced by the c.157G>A mutation or extension of the intron abolishes exon 2 and significantly reduces exon 3 inclusion.

It is becoming increasingly clear that exonic mutations that affect splicing are often incorrectly stratified as missense variants (46, 47). A more comprehensive analysis of such variants will be pivotal to understand their functional outcome, shed new light into the mechanisms of mRNA splicing, and uncover mutations addressable by approved splice modulating therapies like exon skipping. This may be especially relevant for large genes like *DMD* or *DYSF*, where removal of 1 or several exons can result in a truncated yet partially functional protein (48, 49) and could rescue a frameshift induced by aberrant exon exclusion.

In summary, we provide the first evidence to our knowledge that gene-edited human primary MuSC can be harnessed and are a promising and safe source for autologous cell replacement therapies of MD. Routine generation of gene-repaired primary MuSC to use in a clinical setting will require adapting the gene delivery methods and developing pipelines for systematic off-target profiling and biosafety testing. Transient ex vivo ABE expression would evade possible complications associated with preexisting immunity against Cas9 (50). Whether restoring α-sarcoglycan could elicit an immune response requires further investigation. Despite the concerns raised so far for systemic AAV administration, in vivo gene editing has the unique potential of targeting all muscles throughout the body. Provided that further developments guarantee an acceptable risk profile, the benefits might well outweigh the drawbacks. We can, thus, envision future treatments for MD patients combining in vivo gene editing to target the majority of myofibers in affected muscles, as well as cell-based therapies with autologous gene corrected MuSC to ensure a long-term muscle homeostasis by gene repaired muscle stem and progenitor cells. As of now, cell replacement therapies with gene corrected autologous MuSC to restore function in a subset of critical muscles are reachable, are potentially safe, and could substantially improve patients’ life quality and autonomy.

## Methods

### Patients.

Primary MuSC were generated from a 13-year-old male patient with MD due to compound heterozygous mutations in *SGCA*, c.157G>A and c.748-2A>G. The patient has limb girdle weakness but no cardiac failure. Both parents (carriers) are clinically unaffected. All family members are of White. There is 1 sister who is also affected.

### Primary MuSC isolation and culture.

Immediately after the biopsy procedure, the muscle specimen was transferred into Solution A for transport (30 mM HEPES, 130 mM NaCl, 3 mM KCl, 10 mM D-glucose, and 3.2 μM Phenol red, pH 7.6; Carl Roth). The fresh muscle specimen was manually dissected, and fragments were subjected to hypothermic treatment at 4°C–6°C for 2–7 days prior to downstream processing for MuSC isolation (17, 18). Oligoclonal MuSC colonies were obtained following mechanical dissection as described (18). The outgrowing colonies were expanded until passage 4 and characterized prior to cryopreservation. To enhance the probability of available MuSC in difficult-to-handle biopsy specimens, classical purification was performed in parallel (14). All cell populations used in this study were ≥ 95% positive for Desmin. To induce myoblast-to-myotube fusion, medium was switched to Opti-MEM I Reduced Serum Media (Thermo Fisher Scientific) once cells reached confluence.

### iPSC generation and characterization.

Patient iPSC were generated and characterized as described (51). Reagents are listed in Supplemental Table 3. iPSC are available in the Human Pluripotent Stem Cell Registry (hPSCreg; https://hpscreg.eu/cell-line/MDCi017-A) under the cell line identifier MDCi017-A. The hiPSC line with the homozygous c.157G>A mutation was identified in an experiment targeting the *SGCA* locus with CRISPR/Cas9, followed by clonal selection, PCR, and Sanger sequencing to confirm the sequence and zygosity. Based on the SNP signature of both alleles along the entire *SGCA* locus, the homozygous c.157G>A mutation likely results from loss of heterozygosity (LOH) due to an interhomologous recombination event.

### Minigene cloning.

*SGCA* exons 1–4 were amplified from patient or control gDNA. For extension of intron 2, the minigene was amplified in 2 parts using additional primers positioned within intron 2. Approximately 500 bp of the human *HBB* (β-globin) intron 2 were amplified from genomic DNA and joined between the 2 segments of the minigene. All constructs were cloned into pcDNA3.1 digested with HindIII and XhoI. Positive colonies were verified by test digest and Sanger sequencing. Primers are listed in Supplemental Table 2.

### Minigene splicing assay.

Minigenes were transfected into HEK293T cells using Roti-Fect, and cells were harvested after 48 hours. RNA was extracted using RNATri (Bio&Sell), and genomic/plasmid DNA was eliminated via DNaseI digest. RNA (1 μg) was used in a plasmid-specific reverse transcription (RT) reaction. The minigene was analyzed using plasmid-specific primers (Supplemental Table 2) upstream and downstream of the inserts in a standard PCR reaction. The obtained products were resolved on a 2% agarose gel. Low-cycle PCR was performed with a ^32^P-labeled forward primer, and the products were separated by denaturing PAGE. Bands were visualized with a Phosphoimager and quantified using ImageQuantTL. Quantifications are presented as mean ± SD (*n* = 3).

### ABE plasmids.

A plasmid (HE_p4.1) containing a mammalian codon-optimized CAG-driven *S*. *pyogenes* Cas9 (SpCas9) with a double C-terminal nuclear localization signal (52), followed by a T2A-Venus cassette plus a human U6 promoter–driven sgRNA scaffold, was assembled as follows: Cas9-Venus from pU6chimRNA-CAG-Cas9-venus-bpA_(oriA) (Addgene, 86986) was exchanged for Cas9-T2A-Venus from pCAG-Cas9v2T2A-venus-T2A-Kk-bpA (Ralf Kühn lab). Plasmid pCAG-Cas9v2T2A-venus-T2A-Kk-bpA was generated by Gibson assembly from plasmid pCAG-Cas9v2T2A-venus-bpA (Ralf Kühn lab), opened with BsrGI and MluI, and a PCR fragment for the insertion of a T2A peptide and truncated mouse MHC class I molecule H-2Kk was amplified from plasmid pMACS-Kk.II (Miltenyi Biotec) using the primers Ck1 (CTCTCGGCATGGACGAGCTGTACAAGAGGGCAAAGAGGGAGGGCAGAGGAAGTCTTCTAACATGCGGTGACGTGGAGGAGAATCCCGGCCCTATGGCACCCTGCATGCTGCTCC) and Ck2 (CTAGAACTAGTGGATCTGCAacgcgtATTATCACCCTCCTTTTCCACCTGTGTTTC). Plasmid pCAG-Cas9v2T2A-venus-bpA was generated by Gibson assembly using plasmid pCAG-Cas9v2-bpA (Ralf Kühn lab), opened with SphI and MluI, and 2 PCR products for the insertion of a T2A peptide and Venus coding sequence downstream of Cas9 using the primer pairs CV1 (CCACCGTGCGGAAAGTGCTGAG)/CV2 (TCACCGCATGTTAGAAGACTTCCTCTGCCCTCCCTCTTT-GCCCTGTCCACTTTCCGCTTTTTCTTAGGATC) and CV3 (GAAGTCTTCTAACATGCGGTGACGTGGAG-GAGAATCCCGGCCCTATGGTGAGCAAGGGCGAGGAGC)/CV4 (CTTGTACAGCTCGTCCATGCCGA-GAG) together with an overlapping PCR product for the insertion of a polyA signal (bpA) derived from the bovine growth hormone gene using the primer pair CV5 (CGG-CATGGACGAGCTGTACAAGTGATAATacgcgtTGCAGATCCACTAGTTCTAGAGCTCGCTGATCAG)/CV6 (CCATAGAGCCCACCGCATCCCCAG). Plasmid pCAG-Cas9v2-bpA was generated by cloning of a synthetic Cas9 coding region in between the CAG promoter and bpA region. The origi-nal BbsI site of the sgRNA scaffold of pU6chimRNA-CAG-Cas9-venus-bpA_(oriA) (Addgene, 86986) had been previously exchanged to a BplI site for sgRNA cloning using annealed oligos oHE28 and oHE29 (Supplemental Table 2). HE_p4.1 served as backbone to clone ABE7.10_4.1. The TadA heterodimer and the first 247 bp of Cas9 (D10A) were amplified by PCR from pCMV-ABE7.10 (19) with primers containing homology arms for Gibson Assembly. HE_p4.1 was digested with PacI and BglII, which excise the first 247 bp of the Cas9 CDS. The insert was cloned into the digested HE_p4.1 backbone using Gibson Assembly mix (New England Biolabs) for 1 hour at 50°C. For cloning ABE7.10_3.1, the part of Cas9 from amino acid 848–1060 was exchanged for the corresponding part of eSpCas9(1.1) (25) from pCAG-eCas9v2(848_1003_1060A)-bpA (Ralf Kühn lab) using EcoRV and BsmI. For sgRNA cloning, ABE7.10_4.1/ABE7.10_3.1 were digested with BplI, and oligos oHE55 and oHE56 (Supplemental Table 2) were annealed and ligated. Constructs were verified by Sanger sequencing.

### iPSC transfection and sorting.

iPSC were plated 1 day before transfection on culture vessels coated with hESC-grade Matrigel (Corning) at a density of 300,000 cells/9.5 cm^2^ in mTeSR1 medium containing 10 μM Y-27632 2HCl (Selleckchem). They were transfected using Lipofectamine Stem Transfection Reagent (Thermo Fisher Scientific) following manufacturer’s instructions. Medium was replaced before transfection by fresh mTeSR1. Two days after transfection, cells were collected for FACS in PBS containing 50% mTeSR1 (Stemcell Technologies), 0.1 mM EDTA (Thermo Fisher Scientific), 10 μM Y-27632 2HCl (Selleckchem), and 100 μg/mL Primocin (InvivoGen). Venus^+^ cells were sorted using a FACSAria cell sorter (BD Biosciences) (Supplemental Figure 5) and cultured in mTeSR1. Y-27632 2HCl (10 μM) was added to the medium for 2 day or until most iPSC colonies consisted of more than 5–6 cells. Primocin (100 μg/mL) was added to the culture medium for 2 days to prevent contamination.

### Human primary MuSC transfection and sorting.

Human primary MuSC were plated 1 day before transfection at a density of 55,000 cells/9.5 cm^2^ in Skeletal Muscle Cell Growth Medium (SMCGM, Provitro) and transfected using Lipofectamine3000 (Thermo Fisher Scientific) following manufacturer’s instructions. SMCGM was exchanged after 1 day. Two days after transfection, cells were collected for FACS in PBS containing 50% SMCGM, 0.05 mM EDTA, and 100 μg/mL Primocin. Venus^+^ cells were sorted using a FACSAria Fusion cell sorter (BD Biosciences) (Supplemental Figure 5) and cultured in SMCGM. Primocin (100 μg/mL) was added to the culture medium for 2 days.

### Mutation genotyping and genomic editing analysis via EditR.

gDNA was isolated using Agencourt AMPure XP beads (Beckman Coulter). Briefly, each sample was lysed in a heating block with AL-Buffer (QIAGEN) containing 0.2 mg/mL Proteinase K (QIAGEN) for 10 minutes at 56°C. Twice the volume of prewarmed beads was added to each sample and mixed on a rotational wheel. Tubes were placed on a magnetic rack to separate the beads from the supernatant. Beads were washed twice with 80% ethanol, and bound DNA was eluted using FG3 buffer (QIAGEN). Primers oHE24 + oHE25 and oHE26 + oHE27 were used for *SGCA* exon 2 and 7 amplification, respectively (Supplemental Table 2). PCR was performed using Q5 or Phusion High-Fidelity DNA Polymerase (New England Biolabs) and cleanup of PCR products was done with a NucleoSpin Gel and PCR Clean-up kit (Macherey-Nagel). Sequence chromatograms were analyzed with EditR (24).

### Off-target prediction.

We used CRISPOR (53) for off-target prediction. A total of 70 off-target sites is predicted for gRNA#1 with SpCas9 (regardless of whether they contain adenines within the ABE activity window). Only 1 (the WT *SGCA* exon 2 allele) has a single mismatch to the target site, while 3 and 65 sites have, respectively, 3 or 4 mismatches. Of those sites, only 1 contains 3 mismatches located outside the protospacer seed sequence. Most predicted sites are intronic or intergenic, with only 7 sites located in exons. We chose the 4 predicted exonic off-target sites containing adenines within the ABE activity window (protospacer positions 4–8) for further analysis.

### Amplicon sequencing.

Genomic DNA was isolated as described above. The following primers (Supplemental Table 2) were used for the first PCR amplification step: oHE255 + oHE256 (*SGCA*), oHE243 + oHE259 (*ZNF571*), oHE260 + oHE246 (*KIF1A*), oHE261 + oHE248 (*C1orf86*/*FAAP20*), oHE249 + oHE262 (*AJAP1*). Following gel extraction, 20 ng of product from the first amplification step was used for a second amplification step with the same forward and reverse primers as before but containing Illumina adaptor sequences as 5′ overhangs (oHE263-oHE272; Supplemental Table 2). PCR bands were gel extracted, and the DNA concentration was measured using a Qubit fluorometer (Thermo Fisher Scientific). The purity and size of all PCR amplicons was assessed using a 2100 Bioanalyzer system and a DNA 1000 Kit (Agilent). All PCRs were performed using Q5 High-Fidelity DNA Polymerase (New England Biolabs) and cleanup of PCR products was done as above. Amplicon sequencing was performed by GENEWIZ (Amplicon EZ; GENEWIZ Germany GmbH) using an Illumina MiSeq platform with a 2 × 250 bp paired-end read configuration. For *KIF1A*, the PCR amplicon from the first amplification step was submitted to GENEWIZ. Raw sequencing data are available at the NCBI Sequence Read Archive (SRA) under the Bioproject accession no. PRJNA715491 (https://www.ncbi.nlm.nih.gov/sra/PRJNA715491). Amplicon sequencing results were analyzed using CRISPResso2 (26) with the following parameters: Editing tool: Base editors; Sequencing design: Paired end reads; Minimum homology for alignment to an amplicon: 60%; Base editor output: A>G; Center of the quantification window (relative to 3′ end of the provided sgRNA): –10; Quantification window size (bp): 10; Minimum average read quality (phred33 scale): >30; Minimum single bp quality (phred33 scale): No filter; Replace bases with N that have a quality lower than (phred33 scale): No filter; Exclude bp from the left side of the amplicon sequence for the quantification of the mutations: 15 bp; Exclude bp from the right side of the amplicon sequence for the quantification of the mutations: 15 bp.

### RT-PCR and qPCR.

Total RNA was isolated from muscle tissue sections or cultured cells with TRIzol following standard procedures. For RNA isolation from tissue, 1 mL TRIzol was added to 6 × 50 μm cryosections from human muscle biopsies. The suspension was then transferred to 2 mL DNase/RNase free Precellys lysing kit tubes with CK28-R matrix (Bertin Instruments) and homogenized twice for 20 seconds at 6800 rpm using a Precellys homogenizer (Bertin Instruments) before downstream processing for RNA extraction. cDNA synthesis was performed using the QuantiTect Reverse Transcription kit (Qiagen). Q5 High-Fidelity DNA polymerase (New England Biolabs) was used for RT-PCR with primers oHE206-238 (Supplemental Table 2). Cleanup of PCR products was done as above. Relative mRNA levels were quantified via dye-based quantitative PCR (qPCR) in a CFX Connect Real-Time System (Bio-Rad) with primers oHE208-209 and oAK19-31 (Supplemental Table 2). All experiments were performed using KAPA SYBR FAST qPCR Master Mix Universal (Kapa Biosystems). Data were evaluated with the 2^–ΔΔCT^ method. *GAPDH* was used as reference gene. qPCR results were analyzed with Bio-Rad CFX Maestro (v4.1).

### Western blot.

Samples were lysed on ice with lysis buffer (50 mM Tris-HCl, 150 mM NaCl, 0.5% Triton-X100, 0.5% sodium deoxycholate, 1 mM EDTA, 50 mM sodium fluoride, and 1 mM sodium orthovanadate) containing protease inhibitors. Protein concentration was determined using BCA Protein Assay Kit (Thermo Fisher Scientific). For each sample, 20 μg protein diluted in sample buffer (350 mM Tris–HCl, 30% glycerol, 10% sodium dodecyl sulfate, 600 mM DTT, and 0.05% bromophenol blue) were loaded onto a 8%–16% gradient Tris–glycine acrylamide gel (Thermo Fisher Scientific). Blocking was performed with 4% milk powder. Primary antibodies (Supplemental Table 4) were incubated overnight at 4°C, and HRP-conjugated secondary antibodies were incubated at room temperature for 45 minutes. The membrane was incubated with ECL reagent (Thermo Fisher Scientific) and imaged using a VWR CHEMI only system (VWR International GmbH). Images were processed using Adobe Photoshop CS5. Any adaptations were applied to the full image with all lanes. Quantification was performed with ImageJ (NIH).

### Human MuSC transplantation.

*SGCA* c.157Grep patient MuSC that were 99% Desmin^+^, 27% Pax7^+^, 25% Ki-67^+^, 66% MyoD^+^, and 40% Myf5^+^ were used for transplantation. Six-week-old male NOD.Cg-*Prkdc^scid^Il2rg^tm1WjI^*/SzJ (NSG) mice were purchased from Charles River Laboratories 1 week before the experiment. Animal housing and hygienic monitoring followed FELASA recommendations. Focal irradiation of the recipient hind limbs was performed 2 days prior to cell transplantation as described (17, 18). Two injections of 5.5 μL containing 2.5 × 10^4^ cells in a sterile PBS + 2% FCS solution were performed following parallel trajectories into the medial portion of the anterior tibial (TA) muscle (in total, 5 × 10^4^ cells per grafted muscle) as described (18). Mice were sacrificed 19 days after cell transplantation. TA muscles were cryopreserved in liquid nitrogen–chilled isopentane, mounted in gum tragacanth, and stored at –80°C.

### Immunostaining.

Cells cultured on μ-Slides (8-well, ibidi) were fixed with 3.7% formaldehyde, permeabilized with 0.2% Triton X-100, and blocked in 5% bovine serum albumin (BSA)/PBS for 1 hour at room temperature.

Muscle cryosections (6-μm) were cut with a Leica cryostat (CM3050 S). For human-specific Lamin A/C and Spectrin immunostaining, sections were fixed in acetone for 5 minutes at –20°C and blocked with 5% BSA/3% goat serum/PBS for 1 hour at room temperature. For huLaminA/C/Pax7/Laminin-DL488 immunostaining, sections were fixed for 10 minutes at room temperature in 3.7% formaldehyde/PBS, permeabilized with 0.2% Triton-X/PBS for 5 minutes at room temperature, and blocked with 1% BSA/PBS for 1 hour at room temperature. For huSpectrin/α-sarcoglycan immunostaining, sections were fixed in acetone for 5 minutes at –20°C and blocked with 5% BSA/3% goat serum/PBS for 1 hour at room temperature. For huLamin A/C and Desmin immunostaining, sections were fixed for 10 minutes at room temperature in 3.7% formaldehyde/PBS, permeabilized with 0.2% Triton-X/PBS for 5 minutes at room temperature, and blocked with 1% BSA/PBS for 1 hour at room temperature. Cultured cells or muscle cryosections were incubated overnight at 4°C with primary antibodies as indicated (Supplemental Table 4). AlexaFluor 488– or AlexaFluor 568–conjugated secondary antibodies (Thermo Fisher Scientific) were incubated for 2 hours at room temperature. Nuclei were counterstained with Hoechst 33258 (0.5 μg/mL, Sigma-Aldrich). Samples were imaged with a Zeiss LSM 700 confocal microscope (Carl Zeiss MicroImaging GmbH) or with a Leica DMI 6000 fluorescence microscope (Leica Microsystem). Confocal images were composed and edited in ZEN 2.3 (Carl Zeiss Microscopy GmbH) and Adobe Illustrator. For the counting of myogenic and proliferation markers, at least 100 nuclei per sample were counted.

### Statistics.

Details about the statistical tests are described in the corresponding figure legend. Graphs show the mean ± SD where applicable. Group sizes and a description of the represented values and error bars for each figure are indicated in the corresponding figure legend. No samples were excluded from the analysis.

### Study approval.

Research use of human material was approved by the regulatory agencies (EA2/051/10 and EA2/175/17, Charité Universitätsmedizin Berlin), and written informed consent was obtained from donors or legal guardians. Animal experiments were performed under the license number G 0058/20 (LaGeSo).

## Author contributions

HE, AK, and SM designed, conducted, and analyzed experiments. MGDP performed experiments. JK performed MuSC isolation and helped with MuSC characterization. EM helped with iPSC generation and characterization. RK provided vectors and contributed his expertise in gene editing. SK conducted minigene experiments. SK and FH designed and analyzed minigene experiments and discussed splicing data. HE, RK, and SS discussed results. HE and SS designed the study and coordinated the project. HE and SS wrote the manuscript.

## Supplementary Material

Supplemental data

## Version 1 04/13/2021

In-Press Preview

## Version 2 05/24/2021

Electronic publication

## References

[1] Liu W (2019). Estimating prevalence for limb-girdle muscular dystrophy based on public sequencing databases. Genet Med.

[2] Roberds SL (1993). Primary structure and muscle-specific expression of the 50-kDa dystrophin-associated glycoprotein (adhalin). J Biol Chem.

[3] McNally EM (1994). Human adhalin is alternatively spliced and the gene is located on chromosome 17q21. Proc Natl Acad Sci U S A.

[4] Carrié A (1997). Mutational diversity and hot spots in the alpha-sarcoglycan gene in autosomal recessive muscular dystrophy (LGMD2D). J Med Genet.

[5] Alonso-Pérez J (2020). New genotype-phenotype correlations in a large European cohort of patients with sarcoglycanopathy. Brain.

[6] Fendri K (2006). Genetic heterogeneity within a consanguineous family involving the LGMD 2D and the LGMD 2C genes. Neuromuscul Disord.

[7] Trabelsi M (2008). Revised spectrum of mutations in sarcoglycanopathies. Eur J Hum Genet.

[8] Ibraghimov-Beskrovnaya O (1992). Primary structure of dystrophin-associated glycoproteins linking dystrophin to the extracellular matrix. Nature.

[9] Petrof BJ (1993). Dystrophin protects the sarcolemma from stresses developed during muscle contraction. Proc Natl Acad Sci U S A.

[10] Mauro A (1961). Satellite cell of skeletal muscle fibers. J Biophys Biochem Cytol.

[11] Yin H (2013). Satellite cells and the muscle stem cell niche. Physiol Rev.

[12] Lepper C (2011). An absolute requirement for Pax7-positive satellite cells in acute injury-induced skeletal muscle regeneration. Development.

[13] Sambasivan R (2011). Pax7-expressing satellite cells are indispensable for adult skeletal muscle regeneration. Development.

[14] Blau HM (1983). Defective myoblasts identified in Duchenne muscular dystrophy. Proc Natl Acad Sci U S A.

[15] Heslop L (2000). Evidence for a myogenic stem cell that is exhausted in dystrophic muscle. J Cell Sci.

[16] Biressi S (2020). Stem cell therapy for muscular dystrophies. J Clin Invest.

[17] Marg A (2014). Human satellite cells have regenerative capacity and are genetically manipulable. J Clin Invest.

[18] Marg A (2019). Human muscle-derived CLEC14A-positive cells regenerate muscle independent of PAX7. Nat Commun.

[19] Gaudelli NM (2017). Programmable base editing of A•T to G•C in genomic DNA without DNA cleavage. Nature.

[20] Zuo E (2019). Cytosine base editor generates substantial off-target single-nucleotide variants in mouse embryos. Science.

[21] Lee HK (2020). Cytosine base editor 4 but not adenine base editor generates off-target mutations in mouse embryos. Commun Biol.

[22] Yeo G, Burge CB (2004). Maximum entropy modeling of short sequence motifs with applications to RNA splicing signals. J Comput Biol.

[23] Fox-Walsh KL (2005). The architecture of pre-mRNAs affects mechanisms of splice-site pairing. Proc Natl Acad Sci USA.

[24] Kluesner MG (2018). EditR: a method to quantify base editing from sanger sequencing. CRISPR J.

[25] Slaymaker IM (2016). Rationally engineered Cas9 nucleases with improved specificity. Science.

[26] Clement K (2019). CRISPResso2 provides accurate and rapid genome editing sequence analysis. Nat Biotechnol.

[27] Long C (2016). Postnatal genome editing partially restores dystrophin expression in a mouse model of muscular dystrophy. Science.

[28] Nelson CE (2016). In vivo genome editing improves muscle function in a mouse model of Duchenne muscular dystrophy. Science.

[29] Tabebordbar M (2016). In vivo gene editing in dystrophic mouse muscle and muscle stem cells. Science.

[30] Amoasii L (2018). Gene editing restores dystrophin expression in a canine model of Duchenne muscular dystrophy. Science.

[31] Ryu S-M (2018). Adenine base editing in mouse embryos and an adult mouse model of Duchenne muscular dystrophy. Nat Biotechnol.

[32] No authors listed (2020). High-dose AAV gene therapy deaths. Nat Biotechnol.

[33] Gilbert PM (2010). Substrate elasticity regulates skeletal muscle stem cell self-renewal in culture. Science.

[34] Quarta M (2016). An artificial niche preserves the quiescence of muscle stem cells and enhances their therapeutic efficacy. Nat Biotechnol.

[35] Darabi R (2012). Human ES- and iPS-derived myogenic progenitors restore DYSTROPHIN and improve contractility upon transplantation in dystrophic mice. Cell Stem Cell.

[36] Chal J (2015). Differentiation of pluripotent stem cells to muscle fiber to model Duchenne muscular dystrophy. Nat Biotechnol.

[37] Tedesco FS (2012). Transplantation of genetically corrected human iPSC-derived progenitors in mice with limb-girdle muscular dystrophy. Sci Transl Med.

[38] Maffioletti SM (2018). Three-dimensional human iPSC-derived artificial skeletal muscles model muscular dystrophies and enable multilineage tissue engineering. Cell Rep.

[39] Périé S (2014). Autologous myoblast transplantation for oculopharyngeal muscular dystrophy: a phase I/IIa clinical study. Mol Ther.

[40] Richter MF (2020). Phage-assisted evolution of an adenine base editor with improved Cas domain compatibility and activity. Nat Biotechnol.

[41] Gaudelli NM (2020). Directed evolution of adenine base editors with increased activity and therapeutic application. Nat Biotechnol.

[42] Walton RT (2020). Unconstrained genome targeting with near-PAMless engineered CRISPR-Cas9 variants. Science.

[43] Stehlíková K (2014). Autosomal recessive limb-girdle muscular dystrophies in the Czech Republic. BMC Neurol.

[44] Egli D (2018). Inter-homologue repair in fertilized human eggs?. Nature.

[45] Adikusuma F (2018). Large deletions induced by Cas9 cleavage. Nature.

[46] Soukarieh O (2016). Exonic splicing mutations are more prevalent than currently estimated and can be predicted by using in silico tools. PLOS Genet.

[47] Herdt O (2017). The cancer-associated U2AF35 470A>G (Q157R) mutation creates an in-frame alternative 5*′* splice site that impacts splicing regulation in Q157R patients. RNA.

[48] Aoki Y (2012). Bodywide skipping of exons 45-55 in dystrophic mdx52 mice by systemic antisense delivery. Proc Natl Acad Sci U S A.

[49] Malcher J (2018). Exon skipping in a dysf-missense mutant mouse model. Mol Ther Nucleic Acids.

[50] Wagner DL (2019). High prevalence of Streptococcus pyogenes Cas9-reactive T cells within the adult human population. Nat Med.

[51] Metzler E (2020). Generation of two human induced pluripotent stem cell lines derived from myoblasts (MDCi014-A) and from peripheral blood mononuclear cells (MDCi014-B) from the same donor. Stem Cell Res.

[52] Jinek M (2012). A programmable dual-RNA-guided DNA endonuclease in adaptive bacterial immunity. Science.

[53] Concordet J-P, Haeussler M (2018). CRISPOR: intuitive guide selection for CRISPR/Cas9 genome editing experiments and screens. Nucleic Acids Res.

