# Supplementary material for: Base editing repairs an SGCA mutation in human primary muscle stem cells
Source: JCI Insight. 2021 May 24;6(10):e145994. doi: 10.1172/jci.insight.145994 (PMC8262330; doi:10.1172/jci.insight.145994)
Supplement: Supplemental data [file jciinsight-6-145994-s267.pdf]

SUPPLEMETAL DATA

Base editing repairs an *SGCA* mutation in human primary muscle stem cells

Helena Escobar[1]*, Anne Krause[1], Sandra Keiper[2], Janine Kieshauer[1], Stefanie Müthel[1], Manuel García de Paredes[1], Eric Metzler[1], Ralf Kühn[3], Florian Heyd[2], Simone Spuler[1]*.

[1] Muscle Research Unit, Experimental and Clinical Research Center, a joint cooperation of Charité Universitätsmedizin Berlin and Max-Delbrück Center for Molecular Medicine, Berlin, Germany

[2] Freie Universität Berlin, Institute of Chemistry and Biochemistry, Laboratory of RNA Biochemistry, Berlin, Germany

[3] Max-Delbrück Center for Molecular Medicine, Berlin, Germany

*To whom correspondence should be addressed:

Helena Escobar, PhD, Charité Campus Buch, Lindenberger Weg 80, 13125 Berlin, helena.escobar@charite.de; helena.escobar@mdc-berlin.de;

T+49 (0)30 450540523 (Gene editing strategies)

Simone Spuler, M.D., Charité Campus Buch, Lindenberger Weg 80, 13125 Berlin, simone.spuler@charite.de; simone.spuler@mdc-berlin.de;

T+49 (0)30 450540501 (Translational design, muscle stem cells)

SUPPLEMETAL FIGURE LEGENDS

Supplemental Figure 1. Genotyping of *SGCA* c.748-2A>G mutation in control 3. *SGCA* sequence analysis of control 3 shows a heterozygous mutation in the splice acceptor of exon 7 (c.748-2A>G).

Supplemental Figure 2. *SGCA* c.175G>A is an exonic splicing mutation. (**A**) Sequence analysis of the exon 2-3 junction in the full-length (412 bp) RT-PCR band from the c.157G>A carrier from Fig. 2A shows no detectable inclusion of the mutant exon 2. (**B**) RT-PCR analysis of *SGCA* mRNA in human muscle tissue from controls and the heterozygous c.175G>A carrier. The primer binding sites on the *SGCA* coding sequence and the expected band sizes are displayed above. Control 3 is a heterozygous carrier of the *SGCA* c.748-2A>G mutation. Splicing of *SGCA* exons 4-9 is unaffected in the c.157G>A carrier. (**C**) RT-PCR analysis of *SGCA* mRNA in human muscle tissue from controls and the heterozygous c.175G>A carrier was performed using a ^{32}P-labeled forward primer and the products were separated by denaturing PAGE. Splice isoforms were quantified using Phosphoimager analysis. The primer binding sites and expected band sizes are displayed above. fl: splice isoform containing exons 1-5; Δ2: splice isoform missing exon 2; Δ2/3: splice isoform missing exons 2/3.

Supplemental Figure 3. Minigene splicing assays in HEK293T cells. (**A**) Scheme of minigene constructs as in Fig. 2E. (**B**) Splicing pattern of minigene constructs in HEK293T cells analyzed by low-cycle RT-PCR with a ^{32}P-labeled forward primer, products were separated by denaturing PAGE. Quantification is shown in Fig. 2G. (**C**) Splicing pattern of minigene constructs in HEK293T cells analyzed by RT-PCR and agarose gel electrophoresis. The splice isoforms identified were confirmed by Sanger sequencing and are shown on the right. *HPRT* was used as a housekeeping control for RT-PCR analysis. (**D**) Quantification of the splice isoforms from (C). Quantified values are presented as mean ± SD (n=3). *: intron-retention isoforms. 2*: truncated exon 2 (40 nt). 3*: truncated exon 3 (-70 nt). E: exon. I: intron.

Supplemental Figure 4. Generation and characterization of patient iPSC from patient primary myoblasts. (**A**) Immunostaining of iPSC colonies with pluripotency markers. Scale bars: 50 µm. (**B**) Histopathological analysis of iPSC-derived teratomas containing tissues derived from the three germ layers. Scale bars: 50 µm. (**C**) Virtual karyotype analysis of patient iPSC. Regions of gain (duplications) are shown in green, regions of loss (deletions) are shown in red and regions of uniparental disomy (loss of heterozygosity) are shown in grey. Reportable are copy number changes (gains and losses) greater than 0.4Mb compared to the human reference genome and regions of loss of heterozygosity above 3 Mb. (**D**) Histogram plots of flow cytometric analysis of the pluripotency markers SSEA4, Tra1-60, Nanog, Oct3/4, and the differentiation marker SSEA1 in patient iPSC.

Supplemental Figure 5. Gating strategies for FACS-sorting of Venus-positive iPSC and MuSC. Representative FACS plots of primary patient MuSC (**A**) and iPSC (**B**) transfected with ABE7.10_4.1/gRNA#1. Gates were defined to select for the viable cell population (P1) and for doublet exclusion (P2, P3). Venus-positive events (P4) were

separated according to their green fluorescence intensity using stringent gates to ensure a high purity sorting.

Supplemental Figure 6. Adenine base editing in iPSC with a homozygous *SGCA* c.157G>A mutation. (**A**) Experimental design. A plasmid encoding ABE7.10_4.1 or ABE7.10_3.1, a Venus reporter and a U6-driven gRNA expression cassette was transfected into patient iPSC where the c.157G>A mutation had been converted to homozygosis. Venus-positive cells were selected via FACS-sorting and the bulk sorted population was analyzed. (**B**) EditR analysis of nucleotide rates at each protospacer position in patient iPSC transfected with ABE7.10_4.1 and _3.1 in combination with gRNA#1. iPSC transfected with ABE7.10_4.1 without gRNA are shown as control. (**C**) c.157G/A nucleotide rates in EditR analysis from (B). The quantified values are presented as mean ± SD (n=2).

Supplemental Figure 7. Heatmap of A>G conversion rates at the target locus in patient and carrier MuSC. Crispresso2 analysis of amplicon sequencing data showing the percentage of A>G conversion at each position of the quantification window at the target sequence in Venus-positive patient (**A**) and carrier (**B**) MuSC. The target c.157A at protospacer position 6 is indicated in red and the bystander adenine at protospacer position 10 is indicated in orange. A range of concentrations for the ABE7.10_4.1/gRNA#1 vector is shown. Venus-positive cells transfected with the ABE7.10_4.1 vector without gRNA are shown as control.

Supplemental Figure 8. Allele frequencies at the target locus in patient and carrier MuSC. Crispresso2 analysis of allele frequencies in the quantification window at the target sequence in Venus-positive patient (**A**) and carrier (**B**) MuSC. Position c.157 of the *SGCA* CDS is indicated with a red arrow and the bystander adenine at protospacer position 10 is indicated with an orange arrow. The total number and percentage of reads for each allele type is shown on the right. A range of concentrations for the ABE7.10_4.1/gRNA#1 vector is shown. Venus-positive cells transfected with the ABE7.10_4.1 vector without gRNA are shown as control.

Supplemental Figure 9. Heatmap of A>G conversion rates at the predicted off-target loci in patient MuSC. Crispresso2 analysis of amplicon sequencing data showing the percentage of A>G conversion at each position of the quantification window at the four predicted exonic off-target sites (**A to D**) in Venus-positive patient MuSC. Adenines located in the ABE activity window are indicated in orange and mismatched nucleotides between the protospacer sequence and gRNA#1 are indicated with blue stars above. A range of concentrations for the ABE7.10_4.1/gRNA#1 vector is shown. Venus-positive cells transfected with the ABE7.10_4.1 vector without gRNA are shown as control.

Supplemental Figure 10. Allele frequencies at the predicted off-target loci in patient MuSC. Crispresso2 analysis of allele frequencies in the quantification window at the four predicted exonic off-target sites (**A to D**) in Venus-positive patient MuSC. Mismatched nucleotides between the protospacer sequence and gRNA#1 are indicated with blue stars above. The total number and percentage of reads for each allele type is shown on the right. A range of concentrations for the ABE7.10_4.1/gRNA#1 vector is shown. Venus-positive cells transfected with the ABE7.10_4.1 vector without gRNA are shown as control.

Supplemental Figure 11. Donor-derived myofibers express α-sarcoglycan. Grafted muscles were immunostained with antibodies against human Spectrin and α-sarcoglycan. Scale bars: 50 µm.

SUPPLEMENTAL FIGURES

Supplemental Figure 1

Control 3 (carrier *SGCA* c.742-2A>G)

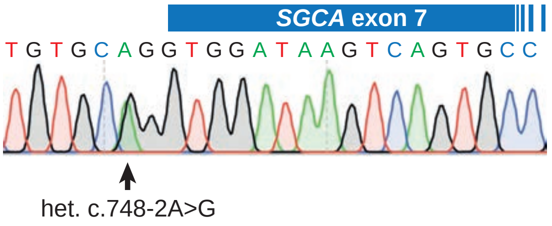

Supplemental Figure 1. Genotyping of *SGCA* c.748-2A>G mutation in control 3. *SGCA* sequence analysis of control 3 shows a heterozygous mutation in the splice acceptor of exon 7 (c.748-2A>G).

Supplemental Figure 2

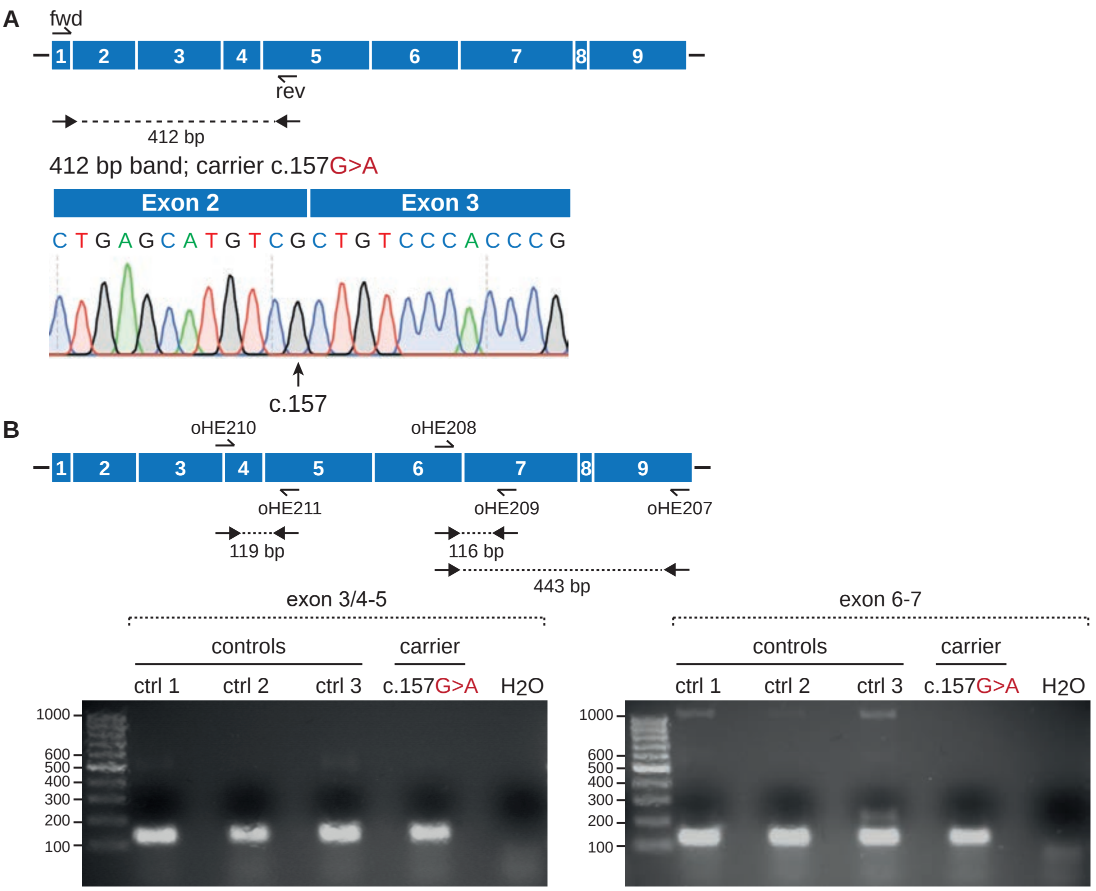

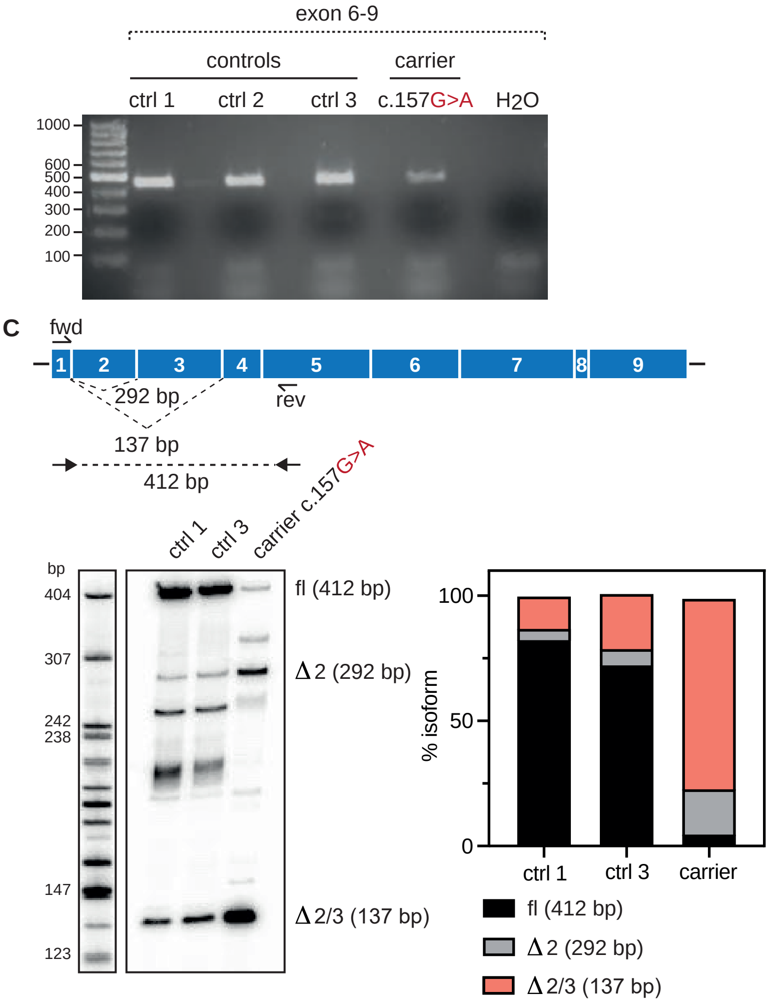

Supplemental Figure 2. *SGCA* c.175G>A is an exonic splicing mutation. (A) Sequence analysis of the exon 2-3 junction in the full-length (412 bp) RT-PCR band from the c.157G>A carrier from Fig. 2A shows no detectable inclusion of the mutant exon 2. **(B)** RT-PCR analysis of *SGCA* mRNA in human muscle tissue from controls and the heterozygous c.175G>A carrier. The primer binding sites on the *SGCA* coding sequence and the expected band sizes are displayed above. Control 3 is a heterozygous carrier of the *SGCA* c.748-2A>G mutation. Splicing of *SGCA* exons 4-9 is unaffected in the c.157G>A carrier. **(C)** RT-PCR analysis of *SGCA* mRNA in human muscle tissue from controls and the heterozygous c.175G>A carrier was performed using a ^{32}P-labeled forward primer and the products were separated by denaturing PAGE. Splice isoforms were quantified using Phosphoimager analysis. The primer binding sites and expected band sizes are displayed above. fl: splice isoform containing exons 1-5; Δ2: splice isoform missing exon 2; Δ2/3: splice isoform missing exons 2/3.

Supplemental Figure 3

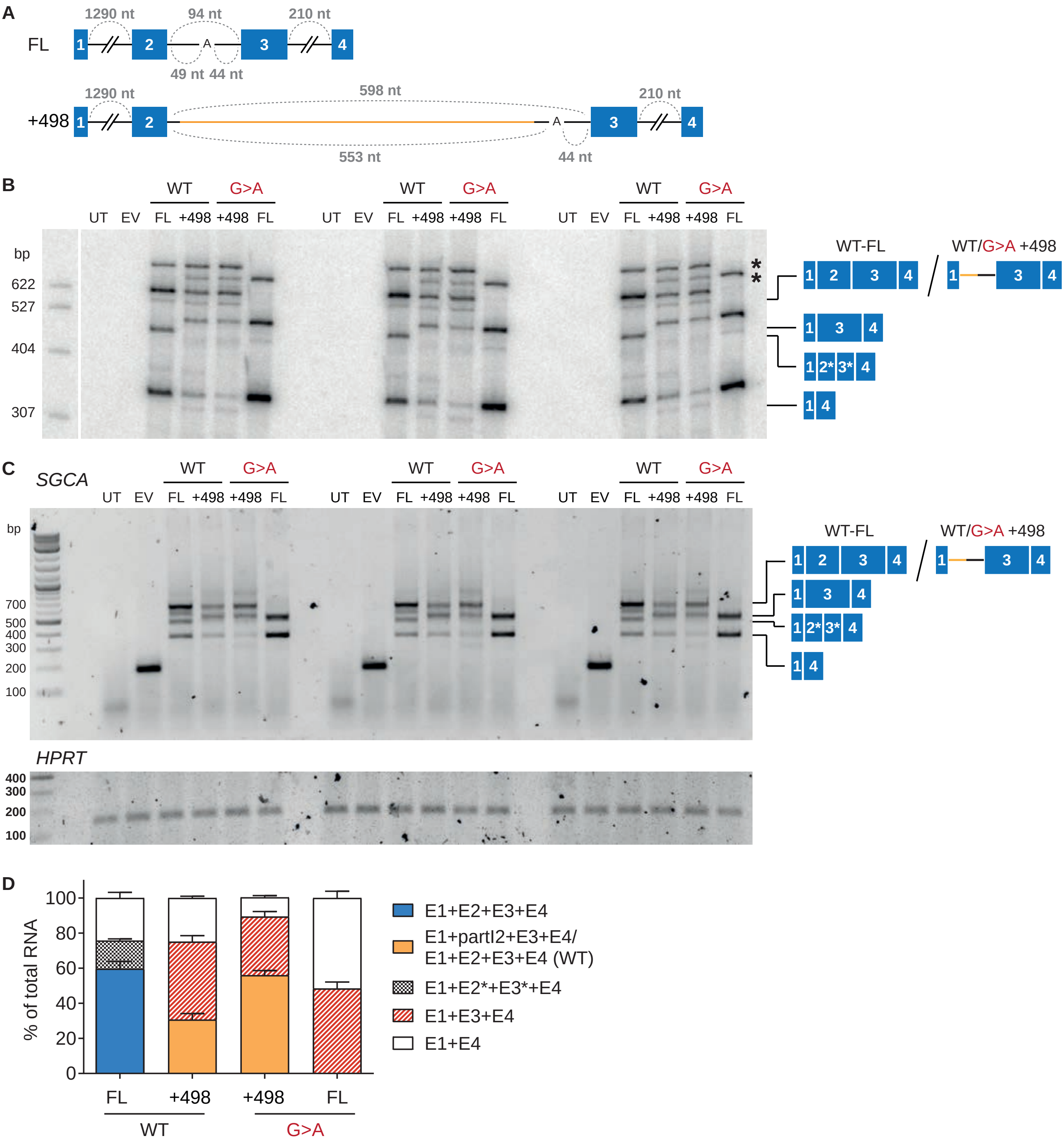

Supplemental Figure 3. Minigene splicing assays in HEK293T cells. (A) Scheme of minigene constructs as in Fig. 2E. **(B)** Splicing pattern of minigene constructs in HEK293T cells analyzed by low-cycle RT-PCR with a ^{32}P-labeled forward primer, products were separated by denaturing PAGE. Quantification is shown in Fig. 2G. **(C)** Splicing pattern of minigene constructs in HEK293T cells analyzed by RT-PCR and agarose gel electrophoresis. The splice isoforms identified were confirmed by Sanger sequencing and are shown on the right. *HPRT* was used as a housekeeping control for RT-PCR analysis. **(D)** Quantification of the splice isoforms from (C). Quantified values are presented as mean ± SD (n=3). *: intron-retention isoforms. 2*: truncated exon 2 (40 nt). 3*: truncated exon 3 (-70 nt). E: exon. I: intron.

Supplemental Figure 4

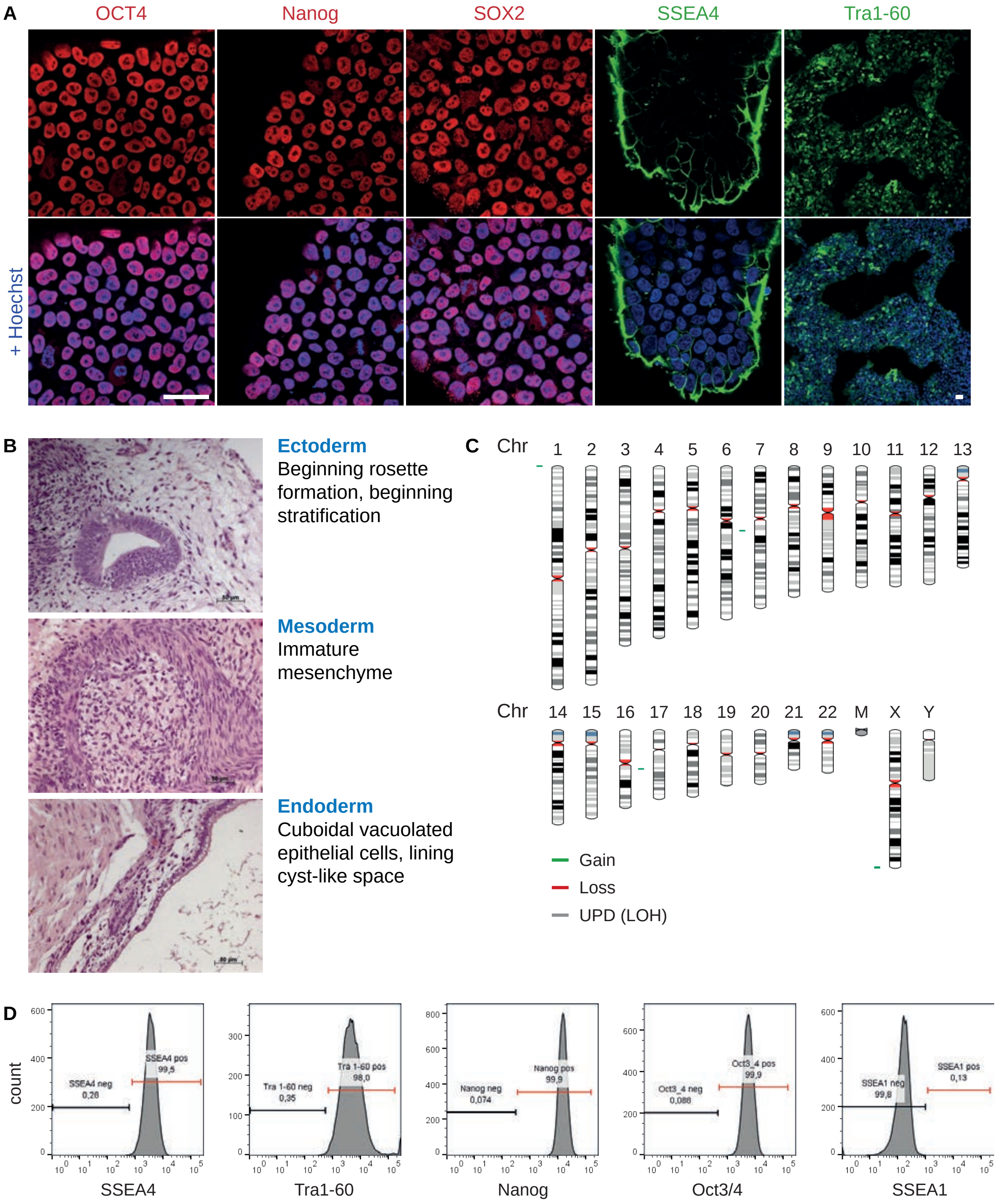

Supplemental Figure 4. Generation and characterization of patient iPSC from patient primary myoblasts. (A) Immunostaining of iPSC colonies with pluripotency markers. Scale bars: 50 µm. **(B)** Histopathological analysis of iPSC-derived teratomas containing tissues derived from the three germ layers. Scale bars: 50 µm. **(C)** Virtual karyotype analysis of patient iPSC. Regions of gain (duplications) are shown in green, regions of loss (deletions) are shown in red and regions of uniparental disomy (loss of heterozygosity) are shown in grey. Reportable are copy number changes (gains and losses) greater than 0.4Mb compared to the human reference genome and regions of loss of heterozygosity above 3 Mb. **(D)** Histogram plots of flow cytometric analysis of the pluripotency markers SSEA4, Tra1-60, Nanog, Oct3/4, and the differentiation marker SSEA1 in patient iPSC.

Supplemental Figure 5

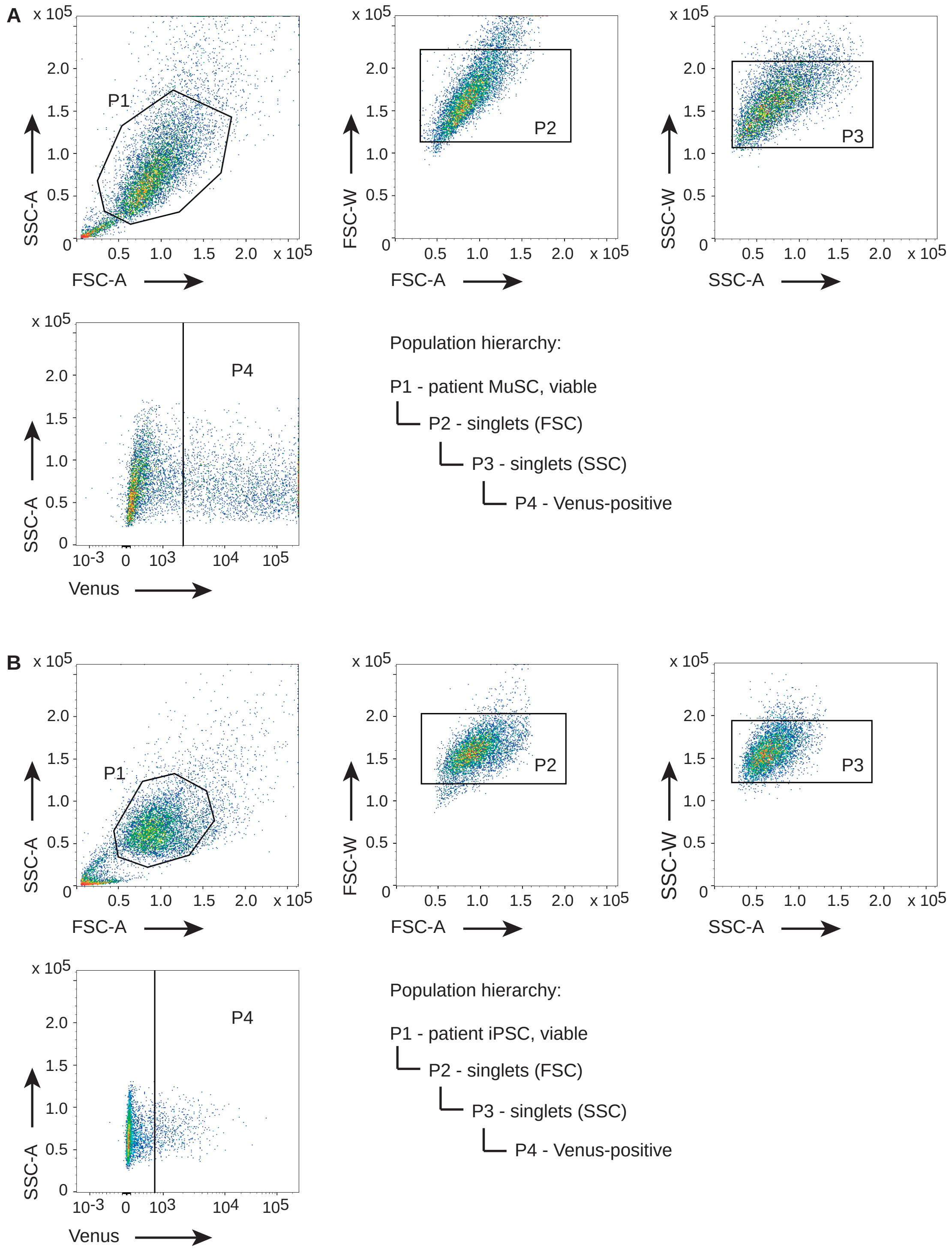

Supplemental Figure 5. Gating strategies for FACS-sorting of Venus-positive iPSC and MuSC. Representative FACS plots of primary patient MuSC **(A)** and iPSC **(B)** transfected with ABE7.10_4.1/gRNA#1. Gates were defined to select for the viable cell population (P1) and for doublet exclusion (P2, P3). Venus-positive events (P4) were separated according to their green fluorescence intensity using stringent gates to ensure a high purity sorting.

Supplemental Figure 6

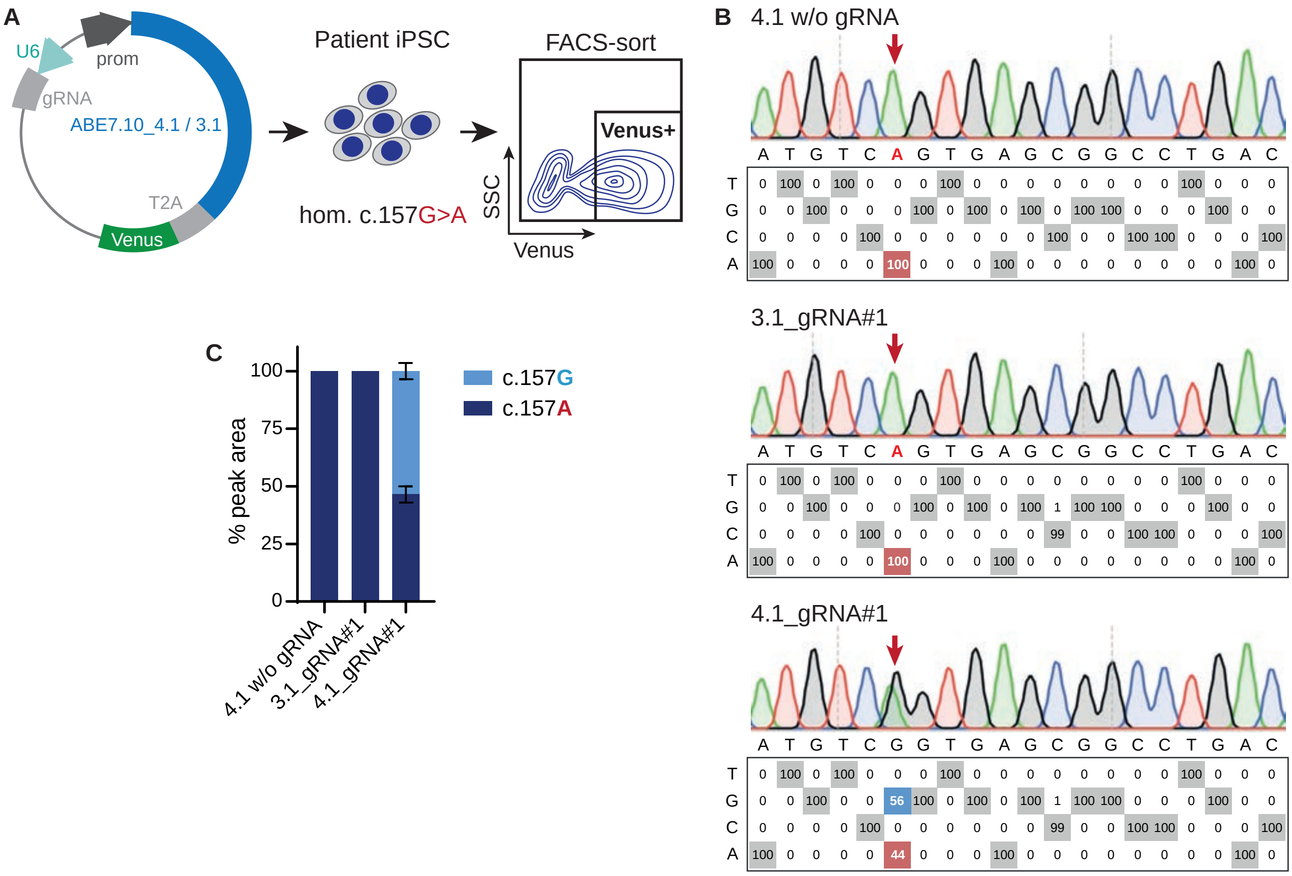

Supplemental Figure 6. Adenine base editing in iPSC with a homozygous *SGCA* c.157G>A mutation. (A) Experimental design. A plasmid encoding ABE7.10_4.1 or ABE7.10_3.1, a Venus reporter and a U6-driven gRNA expression cassette was transfected into patient iPSC where the c.157G>A mutation had been converted to homozygosis. Venus-positive cells were selected via FACS-sorting and the bulk sorted population was analyzed. **(B)** EditR analysis of nucleotide rates at each protospacer position in patient iPSC transfected with ABE7.10_4.1 and _3.1 in combination with gRNA#1. iPSC transfected with ABE7.10_4.1 without gRNA are shown as control. **(C)** c.157G/A nucleotide rates in EditR analysis from (B). The quantified values are presented as mean ± SD (n=2).

Supplemental Figure 7

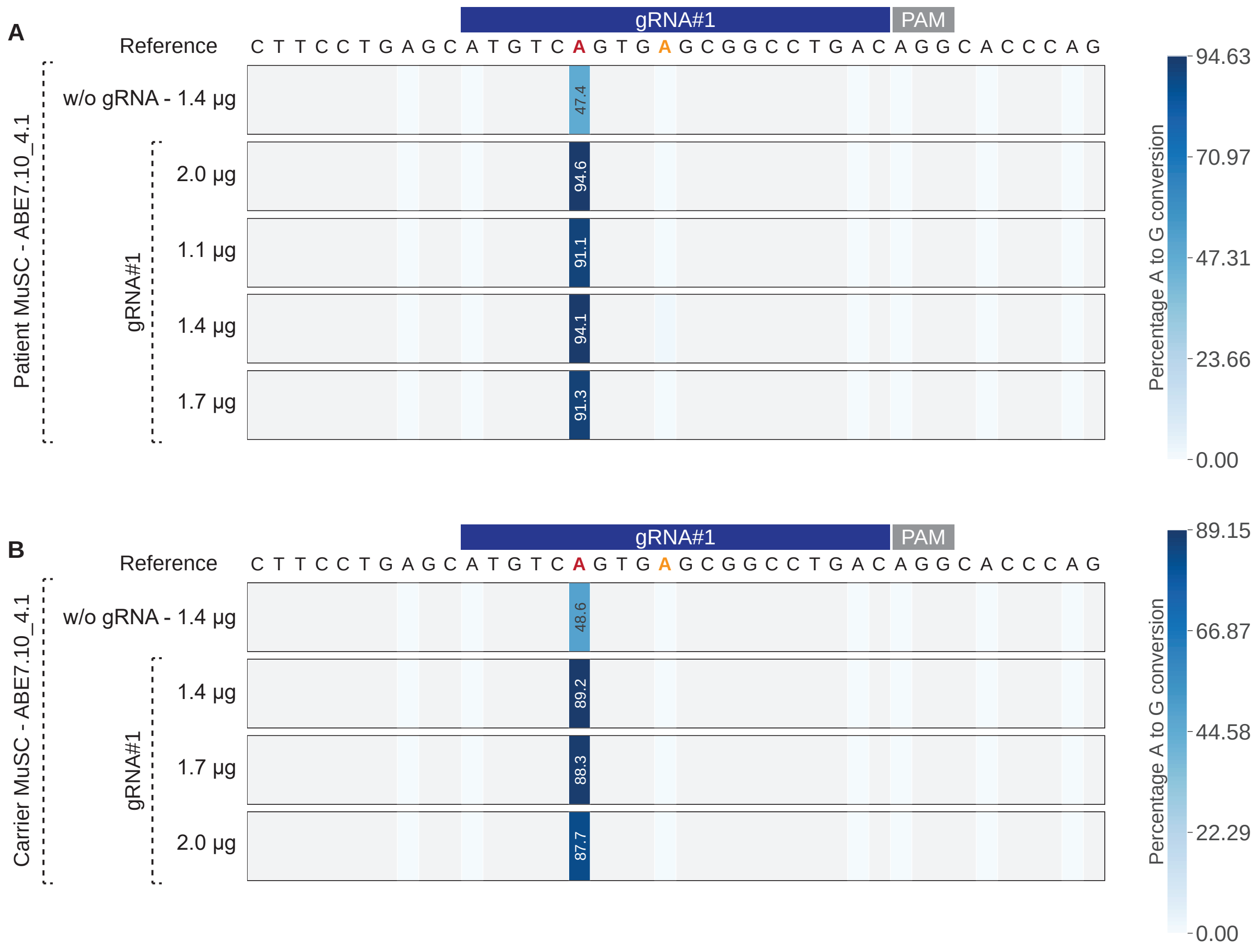

Supplemental Figure 7. Heatmap of A>G conversion rates at the target locus in patient and carrier MuSC. Crispresso2 analysis of amplicon sequencing data showing the percentage of A>G conversion at each position of the quantification window at the target sequence in Venus-positive patient **(A)** and carrier **(B)** MuSC. The target c.157A at protospacer position 6 is indicated in red and the bystander adenine at protospacer position 10 is indicated in orange. A range of concentrations for the ABE7.10_4.1/gRNA#1 vector is shown. Venus-positive cells transfected with the ABE7.10_4.1 vector without gRNA are shown as control.

Supplemental Figure 8

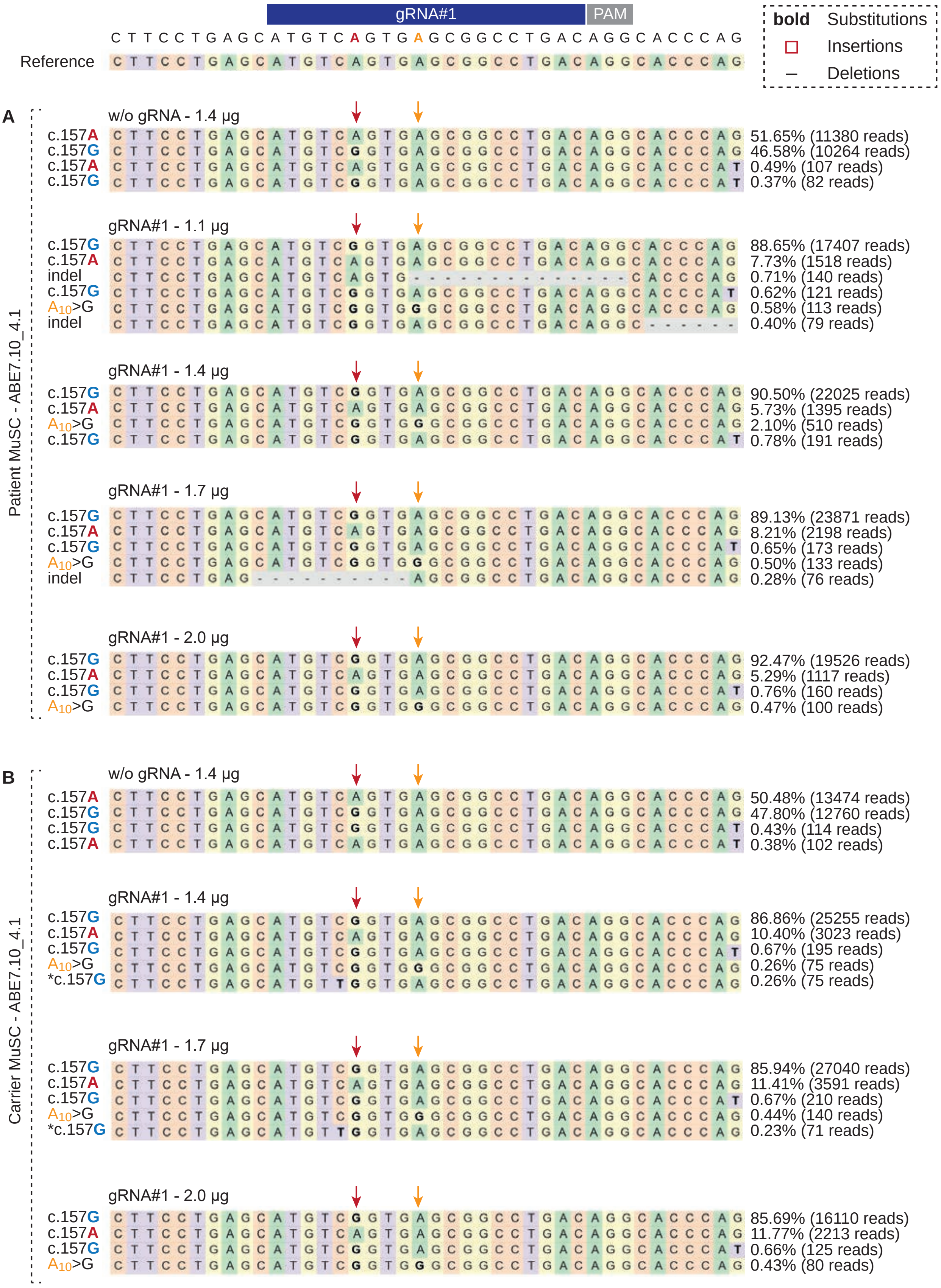

Supplemental Figure 8. Allele frequencies at the target locus in patient and carrier MuSC. Crispresso2 analysis of allele frequencies in the quantification window at the target sequence in Venus-positive patient **(A)** and carrier **(B)** MuSC. Position c.157 of the *SGCA* CDS is indicated with a red arrow and the bystander adenine at protospacer position 10 is indicated with an orange arrow. The total number and percentage of reads for each allele type is shown on the right. A range of concentrations for the ABE7.10_4.1/gRNA#1 vector is shown. Venus-positive cells transfected with the ABE7.10_4.1 vector without gRNA are shown as control.

Supplemental Figure 9

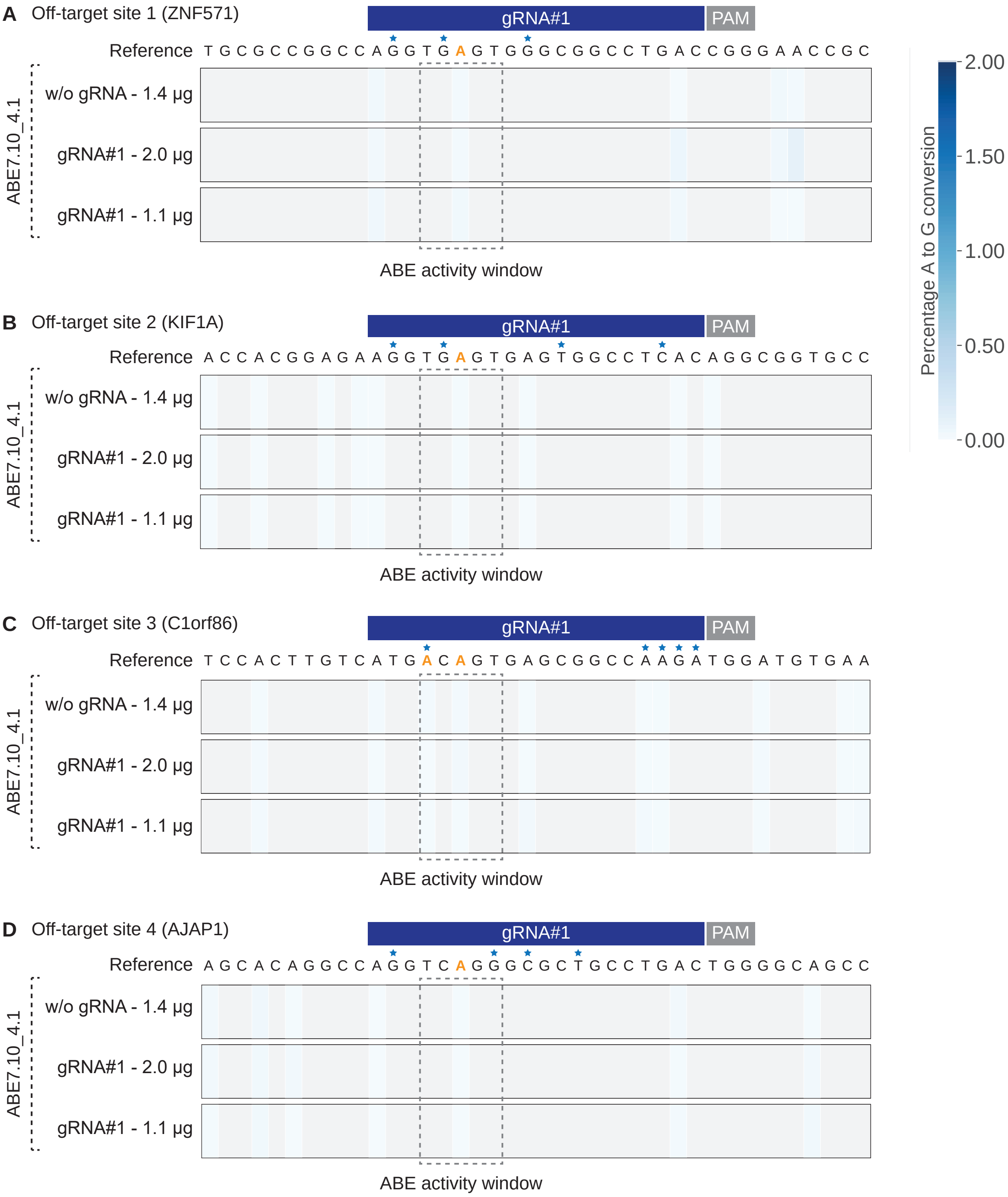

Supplemental Figure 9. Heatmap of A>G conversion rates at the predicted off-target loci in patient MuSC. Crispresso2 analysis of amplicon sequencing data showing the percentage of A>G conversion at each position of the quantification window at the four predicted exonic off-target sites **(A to D)** in Venus-positive patient MuSC. Adenines located in the ABE activity window are indicated in orange and mismatched nucleotides between the protospacer sequence and gRNA#1 are indicated with blue stars above. A range of concentrations for the ABE7.10_4.1/gRNA#1 vector is shown. Venus-positive cells transfected with the ABE7.10_4.1 vector without gRNA are shown as control.

Supplemental Figure 10

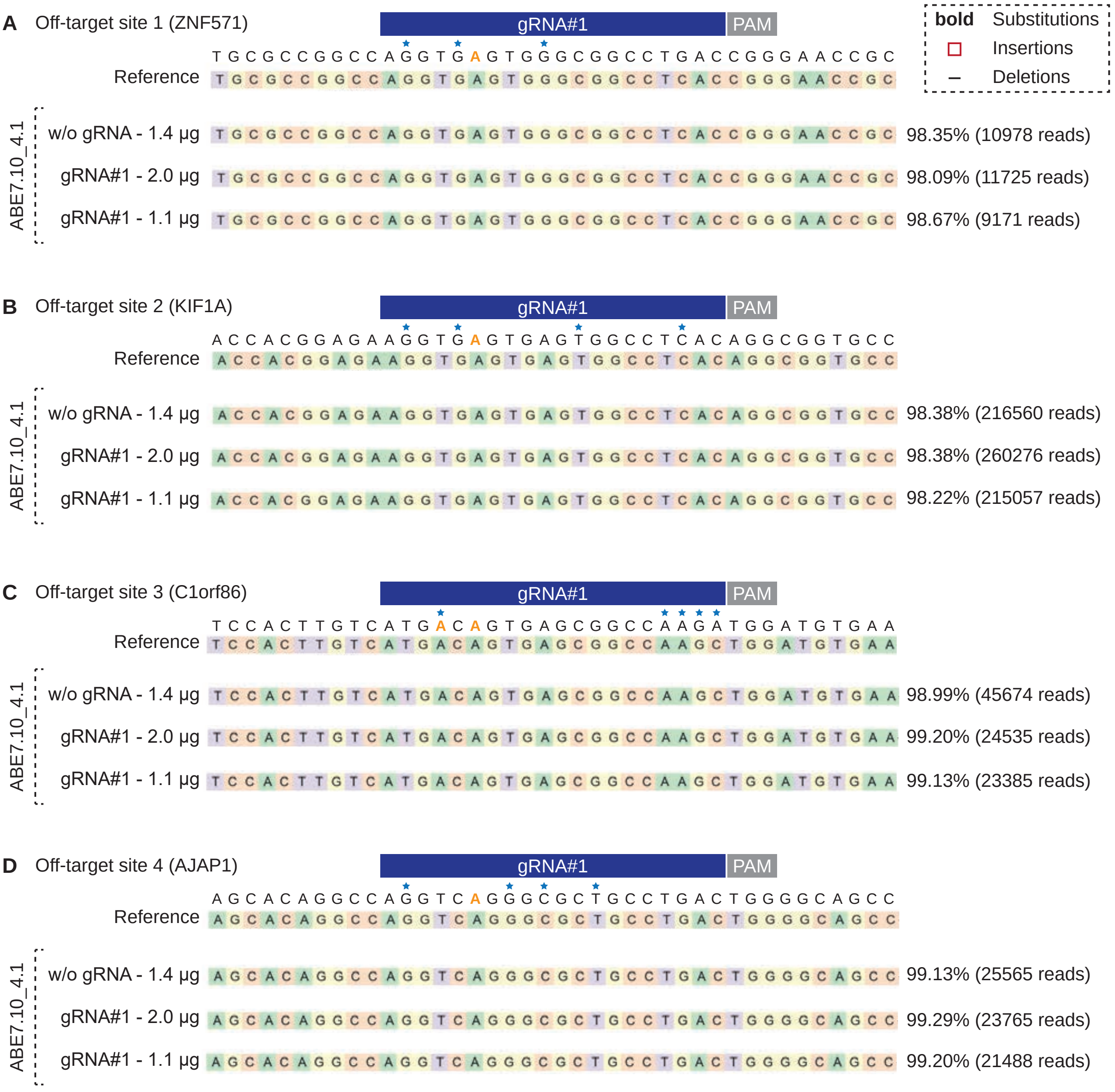

Supplemental Figure 10. Allele frequencies at the predicted off-target loci in patient MuSC. Crispresso2 analysis of allele frequencies in the quantification window at the four predicted exonic off-target sites **(A to D)** in Venus-positive patient MuSC. Mismatched nucleotides between the protospacer sequence and gRNA#1 are indicated with blue stars above. The total number and percentage of reads for each allele type is shown on the right. A range of concentrations for the ABE7.10_4.1/gRNA#1 vector is shown. Venus-positive cells transfected with the ABE7.10_4.1 vector without gRNA are shown as control.

Supplemental Figure 11

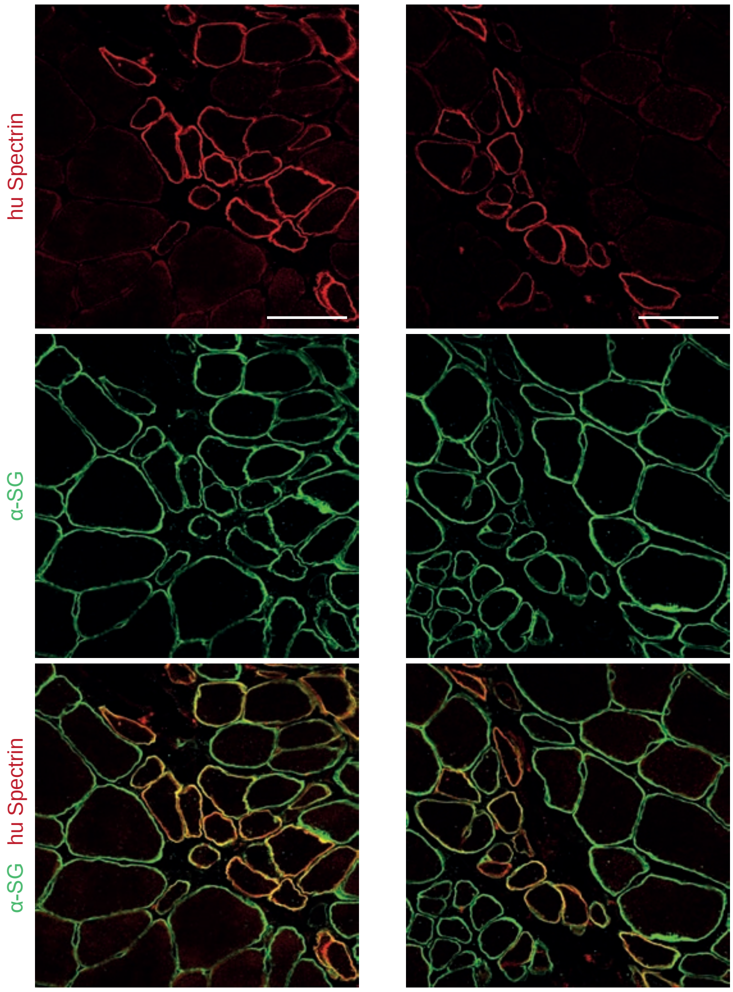

Supplemental Figure 11. Donor-derived myofibers express α-sarcoglycan. Grafted muscles were immunostained with antibodies against human Spectrin and α-sarcoglycan. Scale bars: 50 μm.

SUPPLEMENTAL TABLES

Supplemental Table 1: Myogenic marker expression in *SGCA* c.157Grep patient MuSC

<table>
<tr><th rowspan="2">Patient MuSC populations</th><th rowspan="2" colspan="2">µg vector</th><th colspan="5">% positive cells</th></tr>
<tr><th>Desmin</th><th>Pax7</th><th>Myf5</th><th>MyoD</th><th>Ki67</th></tr>
<tr><td>Unedited</td><td colspan="2">-</td><td>89</td><td>60</td><td>58</td><td>54</td><td>37</td></tr>
<tr><td>ABE7.10_4.1 (w/o sgRNA)</td><td colspan="2">1.4 µg</td><td>97</td><td>13</td><td>46</td><td>71</td><td>37</td></tr>
<tr><td rowspan="6">ABE7.10_4.1/gRNA#1 (SGCA c.175Grep)</td><td colspan="2">1.1 µg</td><td>54</td><td>16</td><td>30</td><td>30</td><td>57</td></tr>
<tr><td rowspan="2">1.4 µg</td><td>cryopreserv.</td><td>99</td><td>27</td><td>40</td><td>66</td><td>25</td></tr>
<tr><td>after recovery</td><td>99</td><td>34</td><td>45</td><td>64</td><td>39</td></tr>
<tr><td rowspan="2">1.7 µg</td><td>cryopreserv.</td><td>98</td><td>20</td><td>53</td><td>44</td><td>39</td></tr>
<tr><td>after recovery</td><td>99</td><td>23</td><td>24</td><td>60</td><td>28</td></tr>
<tr><td colspan="2">2.0 µg</td><td>100</td><td>3</td><td>34</td><td>53</td><td>23</td></tr>
</table>

Supplemental Table 2: Synthetic oligonucleotides for genomic PCR, RT-PCR / RT-qPCR, sequencing and cloning of ABE and minigene vectors

Oligo	Name	Sequence 5' > 3'	Purpose
oHE24	HE_SGCA i1 F	CGCTTCTCTCGGTCCCTTAG	Genotyping *SGCA* c.157G>A mutation / ABE analysis
oHE25	HE_SGCA e3 R	GTAGAGGAAGCCAGGGTGGT	
oHE26	HE_SGCA i6 F	TGGATTCAAACCAGGAGGTC	Genotyping *SGCA* c.748-2A>G mutation
oHE27	HE_SGCA ei7 R	CTTCATGCCCACATTCACCT	
oHE28	BplI-site_oligo1	CACCGGGTCTTCGAGAAGACCTcgtcatcta	exchange of BbsI to BplI site in HE_p4.1
oHE29	BplI-site_oligo2	AAACtagatgacgAGGTCTTCTCGAAGACCC	
oHE55	Sp_sgRNA_*SGCA*ex2mut#1_for	ATGTCAGTGAGCGGCCTGACgtttt	sgRNA#1 cloning into ABE7.10_4.1 and _3.1
oHE56	Sp_sgRNA_*SGCA*ex2mut#1_rev	GTCAGGCCGCTCACTGACATggtgt	
oHE206	SGCA-201_CCDS_ex1 F1	ATGGCTGAGACACTCTTCTG	RT-PCR *SGCA*
oHE207	Sgca_CCDS_ex9 R1	TCAGTGCTGGTCCAGGATAA	
oHE208	SGCA_Sgca_ex6 F1	GCGTTGACTGGTGCAATGT	
oHE209	SGCA_Sgca_ex7 R1	AGTGGGTGGGCAGAAGAAC	
oHE210	SGCA_Sgca_ex3-4 F1	GAGGTCACAGCCTACAATCG	
oHE211	SGCA_Sgca_ex5 R1	TGGCTGCGCACCAGGAACT	
oHE238	SGCA-201_CCDS_ex1 F2	TCTGGACTCCTCTCCTCGTG	
oHE243	ZNF571_Fwd1	GGTGCGGAGGAACACAACTA	ABE OFF-target analysis; primers for
oHE246	KIF1A_Rev1	CACGCCGTCTTCAACATCATC	
oHE248	C1orf86_Rev1	CTCACCAACTGCCCAGATGTG	

oHE249	AJAP1_Fwd1	CCCCGGTTGGTTTGAAAACG	amplicon sequencing
oHE255	SGCA_NGS_ex2 fwd5	TCTTTGTGCACACCTTGGAC	ABE ON-target analysis; primers for amplicon sequencing
oHE256	SGCA_NGS_ex2 rev1	GAGGACTCAGATACCAAATTAGAGG	
oHE259	ZNF571_Rev2	AGTCCCACAGTTCGAGCAAG	ABE OFF-target analysis; primers for amplicon sequencing
oHE260	KIF1A_Fwd2	GGCAGGGGACAGAGTAAGAGA	
oHE261	C1orf86_Fwd2	GCCTGTCCTCTCTGAGTTGC	
oHE262	AJAP1_Rev2	TCAGGTGAGGCTTGTGAGG	
oHE263	SGCA_NGS_ex2 fwd5_ILLUM-ADAPT	ACACTCTTTCCCTACACGACGCTCTTCCGATCTTCTTTGTGCACACCTTGGAC	ABE ON- and OFF-target; primers for amplicon sequencing containing the Illumina adaptares (underlined) as 5'- extension
oHE264	SGCA_NGS_ex2 rev1_ILLUM-ADAPT	GACTGGAGTTCAGACGTGTGCTCTTCCGATCTGAGGACTCAGATACCAAATTAGAGG	
oHE265	ZNF571_Fwd1_ILLUM-ADAPT	ACACTCTTTCCCTACACGACGCTCTTCCGATCTGGTGCGGAGGAACACAACTA	
oHE266	ZNF571_Rev2_ILLUM-ADAPT	GACTGGAGTTCAGACGTGTGCTCTTCCGATCTAGTCCCACAGTTCGAGCAAG	
oHE267	KIF1A_Fwd2_ILLUM-ADAPT	ACACTCTTTCCCTACACGACGCTCTTCCGATCTGGCAGGGGACAGAGTAAGAGA	
oHE268	KIF1A_Rev1_ILLUM-ADAPT	GACTGGAGTTCAGACGTGTGCTCTTCCGATCTCACGCCGTCTTCAACATCATC	
oHE269	C1orf86_Fwd2_ILLUM-ADAPT	ACACTCTTTCCCTACACGACGCTCTTCCGATCTGCCTGTCCTCTCTGAGTTGC	
oHE270	C1orf86_Rev1_ILLUM-ADAPT	GACTGGAGTTCAGACGTGTGCTCTTCCGATCTCTCACCAACTGCCCAGATGTG	

oHE271	AJAP1_Fwd1_ILLUM-ADAPT	ACACTCTTTCCCTACACGACGCTCTTCCGATCTCCCCGGTTGGTTTGAAAACG	
oHE272	AJAP1_Rev2_ILLUM-ADAPT	GACTGGAGTTCAGACGTGTGCTCTTCCGATCTTCAGGTGAGGCTTGTGAGG	
oSS114	ABE_Cas9_BglII_rev	ttggccatctcgttgctgaagatctcttgtaagtaacatattcggttc	Gibson cloning of ABE7.10_4.1
oSS121	ABE+kozak_fwd	ttttcctacagatccttaattaagccgccaccatgtccgaa	
oAK19	SGCA_ex1 F1	GCTGAGACACTCTTCTGGAC	RT-qPCR *SGCA* and *GAPDH*
oAK21	SGCA_ex2 R1	CATGGTCCAAGGTGTGCAC	
oAK23	SGCA_Sgca_ex7 R2	CAGAAGAACGGGTCATGCTC	
oAK30	GAPDH_ex1-Fwd	GAAGGTGAAGGTCGGAGTC	
oAK31	GAPDH_ex3-Rev	GAAGATGGTGATGGGATTTC	
-	MYH2_V1_Fwd	GGAACGGGCTGACATTGCTG	RT-qPCR *MYH2*
-	MYH2_V1_Rev	GTCATTCCATGGCATCAGGACA	
-	HindIII_SGCA_F	AATTTaagcttCCCTGTCTCTGTCACTCACC	Cloning of minigine constructs
-	EcoRI_SGCAPart1_all_R	ATTAgaattcGCCTGTCAGGCCGCTCACCGACAT	
-	EcoRI_SGCAPart1mut_all_R	ATTAgaattcGCCTGTCAGGCCGCTCACTGACAT	
-	EcoRI_Hbbl2_all_F	ATTAgaattcAGTGTGGAAGTCTCAGGATCG	
-	Hbbl2_498_R	GAATGGTGCAAAGAGGCATG	
-	SGCAPart2_498_F	catgcctctttgcaccattcACCCAGGCGGGCGGGCTGGGGTGTA	
-	XhoI_SGCA_R	AATTTctcgagCAAGCCTCTCCTGTCCACAG	
-	T7_seq_F	TAATACGACTCACTATAGGG	PCR & seq., minigene assays
-	BGH_R	TAGAAGGCACAGTCGAGG	

Supplemental Table 3: Reagents for iPSC generation and characterization

**Antibodies used for immunocytochemistry/flow-cytometry**			
	**Antibody**	**Dilution**	**Company & Cat #**
Pluripotency Markers (Immunostaining)	Rabbit anti-OCT4	1:1000	Abcam #ab19857
	Rabbit anti-SOX2	1:300	Abcam #ab97959
	Rabbit anti-NANOG	1:100	Abcam #ab21624
	Mouse anti-TRA-1-60	1:500	Abcam #ab16288
Pluripotency Markers (Flow Cytometry)	Anti-OCT3/4 APC	1:50	Miltenyi Biotec #130-117-709
	Anti-NANOG PE	1:100	Cell Signaling #14955S
	Anti-TRA-1-60 Vio488	1:600	Miltenyi Biotec #130-106-872
	Anti-SSEA4 VioBlue	1:20	Miltenyi Biotec #130-098-366
	Anti-CD15 Vio770	1:100	Miltenyi Biotec #130-113-486

**Primers**		
	**Target**	**Forward/Reverse primer (5′-3′)**
Sendai-virus (RT-PCR)	SeV (total)	GGATCACTAGGTGATATCGAGC / ACCAGACAAGAGTTTAAGAGATATGTATC
	SeV-KOS	ATGCACCGCTACGAGTGAGCGC / ACCTTGACAATCCTGATGTGG
	SeV-KLF-4	TTCCTGCATGCCAGAGGAGCCC / AATGTATCGAAGGTGCTCAA
	SeV-c-Myc	TAACTGACTAGCAGGCTTGTCG / TCCACATACAGTCCTGGATGATGATG
Housekeeping gene (RT-PCR)	Hu18SRNA	GTAACCCGTTGAACCCCATT / CCATCCAATCGGTAGTAGCG

Supplemental Table 4: Antibodies

Antibody	Clone	Company & Cat No.	Working dilution
α-sarcoglycan	EPR14773	Abcam, #ab189254	IF cells/tissue - 1:500; WB – 1:2000
PAX7		Santa Cruz Biotechnology, #sc-81648	IF cells - 1: 200 / tissue - 1: 100
Desmin		Abcam, #ab15200	IF cells - 1:2,000
Desmin		Dako #M0760	IF tissue – 1:50
Ki-67		ThermoFisher Scientifitc, #RM-9106-S0	IF cells - 1:300
MYOD	5.8A	Santa Cruz Biotechnology, #sc-32758	IF cells - 1:50
MYF-5	C20	Santa Cruz Biotechnology, #sc-302	IF cells - 1:2,000
MyHC	MF20	Developmental Studies Hybridoma Bank, #MF20	WB – 1:1000
Skeletal Myosin (FAST)	MY-32	Sigma-Aldrich, #M4276	IF cells - 1:100
Hu Lamin A+C	EPR4100	Abcam, #ab108595	IF tissue - 1:4,000
Hu Spectrin	RBC2/3D5	Leica Biosystems, #NCL-SPEC1	IF tissue - 1:100
Vinculin	VIN-11-5	Sigma-Aldrich, #V4505	WB – 1:200
